# Long non-coding RNA LASSIE regulates shear stress sensing and endothelial barrier function

**DOI:** 10.1038/s42003-020-0987-0

**Published:** 2020-05-26

**Authors:** Laura Stanicek, Noelia Lozano-Vidal, Diewertje Ilse Bink, Aukie Hooglugt, Wenjie Yao, Ilka Wittig, Jos van Rijssel, Jaap Diederik van Buul, Anke van Bergen, Alina Klems, Anne Sophie Ramms, Ferdinand Le Noble, Patrick Hofmann, Robert Szulcek, ShengPeng Wang, Stefan Offermanns, Meryem Seda Ercanoglu, Hyouk-Bum Kwon, Didier Stainier, Stephan Huveneers, Leo Kurian, Stefanie Dimmeler, Reinier Abraham Boon

**Affiliations:** 1 Dept. of Physiology, Amsterdam Cardiovascular Sciences (ACS), Amsterdam UMC, VU University Medical Center, Amsterdam, The Netherlands; 2 Institute of Cardiovascular Regeneration, Center of Molecular Medicine, Goethe-University, Frankfurt, Germany; 3 Department of Medical Biochemistry, Vascular Microenvironment and Integrity, Amsterdam Cardiovascular Sciences (ACS), Amsterdam University Medical Center, 1105 AZ Amsterdam, The Netherlands; 4 Institute for Neurophysiology, Center for Molecular Medicine (CMMC), University of Cologne, Cologne, Germany; 5 Functional Proteomics, SFB 815 Core Unit, Faculty of Medicine, Goethe-University, Frankfurt, Germany; 6 Molecular Cell Biology Laboratory, Department of Plasma Proteins, Sanquin Research and Landsteiner Laboratory, Academic Medical Center Amsterdam, University of Amsterdam, 1066 CX Amsterdam, The Netherlands; 7 Department of Cell and Developmental Biology, Institute of Zoology (ZOO), Karlsruhe Institute of Technology (KIT), Karlsruhe, Germany; 8 German Center for Cardiovascular Research DZHK, Partner Site Frankfurt Rhine-Main, Berlin, Germany; 9 Dept. of Pulmonary Diseases, Amsterdam Cardiovascular Sciences (ACS), Amsterdam UMC, VU University Medical Center, Amsterdam, The Netherlands; 10 Department of Pharmacology, Max Planck Institute for Heart and Lung Research, Bad Nauheim, Germany; 11 Institute of Virology, University Hospital Cologne, 50935 Cologne, Germany; 12 Center for Molecular Medicine Cologne (CMMC), University of Cologne, 50931 Cologne, Germany; 13 Department of Developmental Genetics, Max Planck Institute for Heart and Lung Research, Bad Nauheim, Germany

**Keywords:** Cardiovascular biology, Long non-coding RNAs, Adherens junctions

## Abstract

Blood vessels are constantly exposed to shear stress, a biomechanical force generated by blood flow. Normal shear stress sensing and barrier function are crucial for vascular homeostasis and are controlled by adherens junctions (AJs). Here we show that AJs are stabilized by the shear stress-induced long non-coding RNA LASSIE (linc00520). Silencing of LASSIE in endothelial cells impairs cell survival, cell-cell contacts and cell alignment in the direction of flow. LASSIE associates with junction proteins (e.g. PECAM-1) and the intermediate filament protein nestin, as identified by RNA affinity purification. The AJs component VE-cadherin showed decreased stabilization, due to reduced interaction with nestin and the microtubule cytoskeleton in the absence of LASSIE. This study identifies LASSIE as link between nestin and VE-cadherin, and describes nestin as crucial component in the endothelial response to shear stress. Furthermore, this study indicates that LASSIE regulates barrier function by connecting AJs to the cytoskeleton.

## Abstract (web summary)

Stanicek et al identify a shear stress-induced long non-coding RNA they name LASSIE, which stabilises junctions between endothelial cells through interactions with junctional and cytoskeletal proteins. This study provides insights into how a transcript that does not encode a protein controls endothelial response to forces associated with blood flow and endothelial barrier function.

## Introduction

Blood vessels are lined by endothelial cells (ECs) forming a physical barrier that separates blood from the surrounding tissue. The maintenance of EC-cell junctions is thus crucial for endothelial homeostasis and consequently requires tight regulation and continuous adaptation. Hemodynamic shear stress—a biomechanical force generated by blood flow—is a main regulator of this process. ECs exposed to laminar shear stress (LSS) display transcription of atheroprotective genes, many of these are dependent on Kruppel-like factor 2 (KLF2) and 4 (KLF4)^1–3^. Concurrently, high LSS promotes EC alignment and strengthens EC-cell junctions^4^, thus contributing to endothelial barrier function^5,6^. Aberrant interactions between ECs cause increased permeability and tissue edema, a common cause of many pathological conditions^7,8^.

EC junctions comprise tight junctions (TJs), adherens junctions (AJs) and gap junctions. TJs and AJs contribute to endothelial barrier integrity^9,10^. Both junctional structures are formed between neighboring cells by homophilic assembly of transmembrane proteins that in turn are anchored to the cytoskeleton via intracellular adaptor proteins. Vascular endothelial (VE)-cadherin as main component of AJs directly interacts with β- and γ-catenin (also called junctional plakoglobin). Both catenins bind α-catenin which is responsible for AJs stabilization by associating to actin^11–13^. Another adhesion protein present in EC junctions is platelet endothelial cell adhesion molecule (PECAM-1). Like VE-cadherin, PECAM-1 binds to catenins, namely β- and γ-catenin, and might exert similar biological functions^14^. Moreover, VE-cadherin and PECAM-1 have been reported to form a mechanosensory complex together with VE growth factor receptor 2 that is responsible for shear stress sensing in ECs^15^. Besides actin, intermediate filaments (IF) represent cytoskeletal structures that are described to be involved in that process^16^. Microtubules in contrast have been described as cytoskeletal component indispensable for normal endothelial barrier function^17^. However, the purpose of the various connections of junction proteins to the cytoskeleton is not fully understood.

A vast majority (80%) of the human genome is transcribed into noncoding RNA while only 2–3% codes for proteins, as reported by the encyclopedia of DNA elements project^18^. Noncoding RNAs are frequently classified according to their size into small (<200 nt) and long noncoding RNAs (lncRNAs, >200 nt)^19^. A well-studied example of small noncoding RNAs is the microRNAs. In contrast, the larger long noncoding RNAs are more poorly characterized. Yet, several studies propose diverse mechanisms of action in a wide range of biological processes. The ability of lncRNAs to bind proteins and nucleic acids enables them to act as guidance molecules. One example is the involvement of lncRNAs in the regulation of gene expression by recruiting chromatin modifiers or transcription factors^20,21^. Many lncRNAs are exported from the nucleus and exert their function posttranscriptionally in the cytoplasm or specific organelles, where they are known to regulate mRNA stability and protein translation^22,23^.

LncRNAs were previously shown to exert regulatory functions in the endothelium, while several lncRNAs have emerged as epigenetic regulators in ECs^24–27^. Nonetheless, the involvement of lncRNA transcripts in the regulation of shear stress sensing in ECs remains unexplored. In this study, we identify the shear stress-induced lncRNA LASSIE (LncRNA activated by shear stress in the endothelium) and characterize its role in EC function. We show that LASSIE stabilizes EC junctions through interaction with junctional and cytoskeletal proteins to promote the association of the cytoskeleton to AJs. LASSIE plays a crucial role in junction stability and barrier function in ECs and is indispensable for normal shear stress sensing.

## Results

### LASSIE is a shear stress-induced lncRNA

**Fig. 1:**
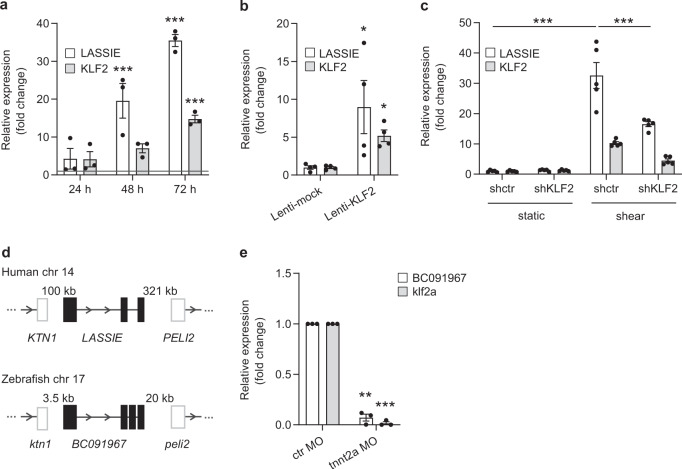
LASSIE is a shear stress-induced lncRNA. **a**, **b** HUVECs were exposed to laminar shear stress (20 dyn/cm^2^) or cultured at static condition. Changes in LASSIE and KLF2 expression by different types of shear stress were assessed by qRT-PCR. Expression values are relative to static condition and normalized to GAPDH mRNA. KLF2 is shown as a shear stress-induced positive control. **a** Cells were exposed to shear stress for the indicated time periods (*n* = 3; two-way ANOVA; static 48 h vs. shear 48 h: *p* < 0.0001 (LASSIE); static 72 h vs. shear 72 h: *p* < 0.0001 (LASSIE); *p* = 0.0001 (KLF2)). Gray line depicts static control levels. **b** The effect of lentiviral-mediated (Lenti) KLF2 induction on LASSIE expression in static HUVECs was analyzed by qRT-PCR. Expression is relative to mock-transduced cells and normalized to RPLP0 mRNA (*n* = 4; Mann–Whitney test; LASSIE: *p* = 0.0286; KLF2: *p* = 0.0286). **c** KLF2-dependent expression of LASSIE was analyzed by lentiviral-mediated shRNA knockdown of KLF2 in HUVECs exposed to laminar shear stress (20 dyn/cm^2^ for 72 h), RNA levels were assessed by qRT-PCR. Expression is relative to mock-transduced cells and normalized to GAPDH mRNA (*n* = 5; two-way ANOVA, LASSIE: shctr static vs. shctr shear: *p* < 0.0001; shctr shear vs. shKLF2 shear: *p* < 0.0001). **d** The *KTN1-LASSIE-PELI2* locus is conserved between human and zebrafish. **e** Fli1a:EGFP embryos were injected with 4 ng tnnt2a and control (ctr) morpholino (MO) to asses shear stress-dependent expression of zebrafish *LASSIE* (*BC091967*). Seventy-two hours postfertilization GFP positive cells were sorted by FACS and subsequently analyzed by qRT-PCR. Expression values are relative to ctr MO treated zebrafish. klf2a is shown as a shear stress-induced positive control, expression is normalized to elf1a mRNA (*n* = 223 (ctr MO) *n* = 234 (tnnt2a MO) over three independent experiments; one-sample *t*-test: klf2a: *p* = 0.0002 BC091967: *p* = 0.014) (*p < 0.05; **p < 0.01; ***p < 0.001).

Using previously published RNA sequencing analysis of human umbilical vein endothelial cells (HUVECs) exposed to LSS (20 dyn/cm^2^ for 72 h) and cultured at static condition^28^, we identified the lncRNA linc00520, exhibiting a 34-fold induction in shear stress-exposed HUVECs compared with static cells, as validated by qRT-PCR (Fig. 1a). The noncoding potential of linc00520 was confirmed by comparison with the directly upstream and downstream located protein coding genes *PELI2* and *KTN1* using the computational prediction tool CPAT^29^ (Supplementary Fig. 1a). This lncRNA is expressed in a wide range of ECs isolated from different vascular beds (Supplementary Fig. 1b) and was subsequently termed LASSIE, given its strong and consistent induction by prolonged LSS (Fig. 1a). In contrast, LASSIE expression is not significantly affected by oscillatory shear stress, as compared with static conditions (Supplementary Fig. 1c). Furthermore, LASSIE expression is induced by shear stress in different vascular ECs, such as microvascular ECs, pulmonary arterial ECs, and aortic ECs, as well as by different shear stress magnitudes (Supplementary Fig. 1d–g). The role of the transcription factor KLF2 in LASSIE expression was analyzed, as KLF2 is a known inducer of many shear stress-responsive genes in ECs^1,2^. Lentiviral overexpression of KLF2 in static conditions resulted in a ninefold upregulation of LASSIE (Fig. 1b). Furthermore, silencing of KLF2 using short hairpin RNA diminishes the induction of LASSIE in LSS-exposed HUVECs (Fig. 1c). These results demonstrate a partly KLF2-dependent expression of LASSIE upon exposure to LSS.

Next, we aimed to identify functional homologues of LASSIE in vivo. The genomic LASSIE sequence is poorly conserved between human, mouse, and zebrafish. The zebrafish *BC091967* and the human *LASSIE* gene share a homologous locus and a similar exon architecture (Fig. 1d). Thus, the functional conservation of this gene was addressed by assessing shear stress responsiveness in zebrafish. To this end, morpholinos targeting cardiac troponin T2 (Tnnt2) were used in zebrafish that consequently lack blood flow, as previously described^30^. We used fli1a:EGFP zebrafish that express EGFP in ECs and separated ECs from non-ECs by FACS-sorting. ECs from Tnnt2a morphants exhibited greatly reduced expression of BC091967 and klf2a compared with control morphants (Fig. 1e, Supplementary Fig. 2). These results show that the zebrafish transcript *BC091967* from the locus homologous to human LASSIE is shear stress responsive as well.

### LASSIE regulates endothelial cell function

**Fig. 2:**
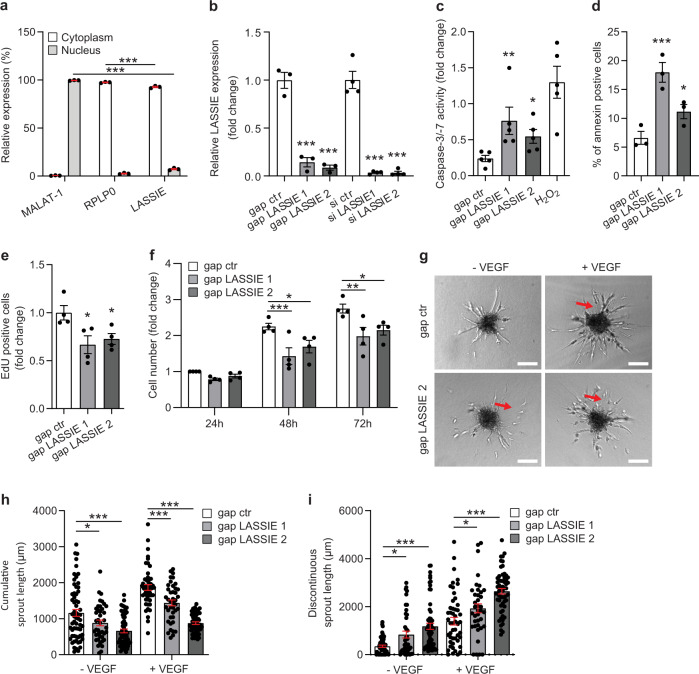
LASSIE regulates endothelial cell function. **a** Distribution of LASSIE was analyzed by qRT-PCR in nuclear and cytoplasmic fractions of HUVECs, MALAT-1 is shown as a nuclear enriched lncRNA and RPLP0 as a cytoplasmic enriched mRNA (*n* = 3; one-way ANOVA; nuclear fraction: MALAT-1 vs. LASSIE: *p* < 0.0001; cytoplasmic fraction: RPLP0 vs. LASSIE: *p* < 0.0001). **b**–**g** HUVECs were transfected with GapmeR (gap) or siRNA (si) targeting LASSIE or a respective control (ctr) sequence, subsequent experiments were performed 48 h post transfection. **b** Relative expression of LASSIE was assessed by qRT-PCR, expression is relative to control cells and normalized to RPLP0 mRNA (siRNA: *n* = 4; gapmeR: *n* = 3, one-way ANOVA; all depicted comparisons: *p* < 0.0001). **c**, **d** Apoptosis was assessed by caspase-3/7 activity relative to control cells (H_2_O_2_ is shown as a positive control; *n* = 5; one-way ANOVA; compared with gap ctr: *p* = 0.0075 (gap LASSIE 1) *p* = 0.0324 (gap LASSIE 2)) and flow cytometry of annexin V stained cells (*n* = 3; one-way ANOVA; compared with gap ctr: *p* < 0.0001 (gap LASSIE 1); *p* = 0.0285 (gap LASSIE 2)). **e**, **f** Proliferation was determined by EdU incorporation (*n* = 4; one-way ANOVA; compared with gap ctr: *p* = 0.0257 (gap LASSIE 1); *p* = 0.0321 (gap LASSIE 2)) and cell counting at the indicated time points post transfection (*n* = 4; two-way ANOVA; compared with gap ctr; 48 h: *p* = 0.0009 (gap LASSIE 1); *p* = 0.023 (gap LASSIE 2); 72 h: *p* = 0.0018 (gap LASSIE 1); *p* = 0.0141 (gap LASSIE 2)). Both experiments are shown relative to control cells. **g** In vitro sprouting was analyzed under basal conditions and VEGF (50 ng/ml) stimulation. Representative images are shown, red arrows indicate discontinuous sprouts. Scale bars are 100 µm (Six independent experiments with more than four spheroids per group were analyzed). **h**, **i** Quantification of cumulative and discontinuous sprout length measurements (two-way ANOVA, compared with gap ctr; *n* = 62 (gap ctr), *n* = 41 (gap LASSIE 1), *n* = 63 (gap LASSIE 2), *n* = 49 (gap ctr +VEGF), *n* = 41 (gap LASSIE 1 +VEGF), *n* = 57 (gap LASSIE 1 +VEGF); cumulative: −VEGF: *p* = 0.0258 (gap LASSIE 1); *p* < 0.0001 (gap LASSIE 2); +VEGF: *p* = 0.0005 (gap LASSIE 1); *p* < 0.0001 (gap LASSIE 2); discontinuous: −VEGF: *p* = 0.0236 (gap LASSIE 1); *p* < 0.0001 (gap LASSIE 2); +VEGF: *p* = 0.0195 (gap LASSIE 1); *p* < 0.0001 (gap LASSIE 2)) (*p < 0.05; **p < 0.01; ***p < 0.001).

To determine the functional role of LASSIE in ECs, we performed loss-of-function experiments in cells. Nuclear–cytoplasmic fractionation revealed a predominant cytoplasmic localization of LASSIE when compared with nuclear enriched lncRNA MALAT-1^31^ and cytoplasmic enriched protein-coding mRNA ribosomal protein lateral stalk subunit P10 (RPLP0) (Fig. 2a). Two different knockdown strategies were applied using locked nucleic acid (LNA) GapmeRs and siRNAs. These oligonucleotides were designed according to LASSIE transcript characterization by 5′ and 3′ RACE (rapid amplification of cDNA ends) (Supplementary Fig. 3a). Both knockdown strategies resulted in a significant reduction of total LASSIE levels by more than 80% (Fig. 2b). The functional role of LASSIE was subsequently analyzed by several in vitro assays. Silencing of LASSIE induced apoptosis as assessed by caspase-3/7 activity and annexin V binding (Fig. 2c, d, Supplementary Fig. 3b), both indicators for apoptosis. Decreased proliferation of LASSIE-silenced HUVECs was observed by determining EdU incorporation and cell counting at distinct time points after transfection (Fig. 2e, f). In contrast, cell migration was not significantly affected (Supplementary Fig. 3c–e). Concomitantly, angiogenic spouting of LASSIE-silenced HUVECs was disturbed, demonstrated by a decrease in total sprout outgrowth and an increase in discontinuous sprout formation, both under basal condition and after stimulation with VEGF (Fig. 2g–i). Impaired angiogenic sprouting due to insufficient stalk cell function in LASSIE-silenced ECs implies a crucial impact of this lncRNA on EC function, likely involving cell–cell interactions or cell survival.

### LASSIE interacts with proteins of endothelial junctional complexes

**Fig. 3:**
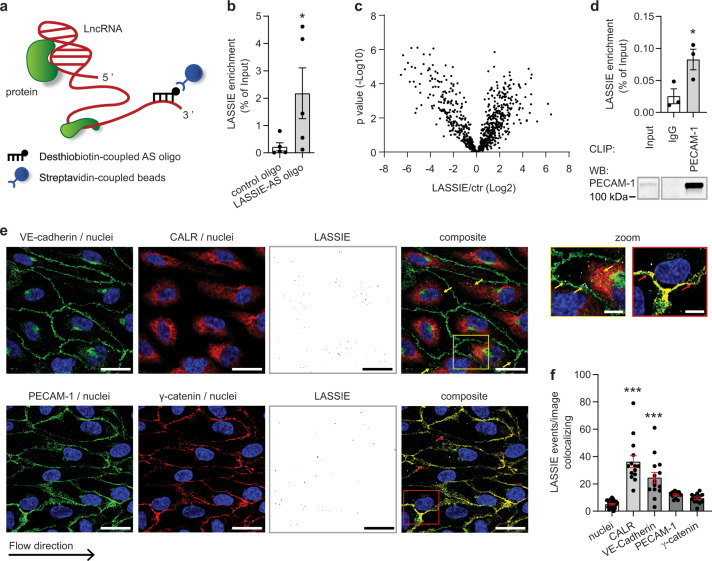
LASSIE interacts with proteins of endothelial junctional complexes. **a** Schematic depiction of the antisense (AS) affinity purification of endogenous RNA-protein complexes. **b** Endogenous LASSIE-protein complexes were captured by RNA-antisense purification. LASSIE was targeted by a desthiobiotin-coupled 2′O-Me-RNA-antisense oligonucleotide (AS) by incubation with HUVEC cell lysates. RNA-protein complexes were captured using streptavidin beads. Biotin elution and subsequent qRT-PCR detected LASSIE enrichment compared with a control desthiobiotin-coupled 2′O-Me-RNA oligonucleotide, enrichment is shown as percentage of the input fraction (*n* = 5; ratio paired *t-*test; *p* = 0.0315). **c** Biotin elutions from LASSIE-antisense purification were analyzed by mass spectrometry (*n* = 5). **d** HUVEC cell lysates were incubated with an anti-PECAM-1 antibody and isotype control (IgG) for crosslinking RNA immunoprecipitation (CLIP). Enrichment of co-purified LASSIE RNA was detected by qRT-PCR and shown as percentage of the input fraction (*n* = 3; ratio paired *t*-test: *p* = 0.0332). CLIP efficiency was analyzed by western blotting (WB) with an anti-PECAM-1 antibody, 1% of the lysate was used as a control and was detected separately. Representative WB is depicted. **e** Subcellular localization of LASSIE (white) was analyzed by ViewRNA^®^ in situ hybridization in HUVECs exposed to laminar shear stress (20 dyn/cm^2^ for 72 h). Cells were immunostained for VE-cadherin (green), PECAM-1 (green), the ER marker CALR (red), and γ-catenin (red). Nuclei were labeled with DAPI (blue). Representative images are shown. Scale bars are 25 µm. Arrows indicate LASSIE co-localization with the ER (pointing to the bottom left) and the membrane (pointing to the top right). Boxes correspond to zoomed images of the respective composite image. Scale bars are 5 µm. **f** Co-localization of LASSIE with co-stained proteins was analyzed for DAPI (*n* = 27), CALR (*n* = 14; *p* < 0.0001), VE-cadherin (*n* = 14; *p* < 0.0001), PECAM-1 (*n* = 13), and γ-catenin (*n* = 13) from one experiment (one-way ANOVA; compared with nuclei) (*p < 0.05; **p < 0.01; ***p < 0.001).

Next, we analyzed the underlying molecular mechanism causing the observed biological phenotype. As LASSIE does not greatly influence global gene expression (Supplementary Fig. 4a), we aimed to identify putative protein interaction partners by RNA-antisense (AS) purification. To this end, LASSIE accessibility was examined by RNase H digestion and oligonucleotide binding was subsequently validated by qRT-PCR, as previously described^27^ (Supplementary Fig. 4b, c). Using a 3′ desthiobiotin-TEG labeled 2′ O-Me-RNA LASSIE-AS oligonucleotide yielded a ninefold enrichment of LASSIE compared with a non-targeting control oligonucleotide (Fig. 3a, b). LASSIE was purified by streptavidin pulldown from HUVECs lysate, using physiological isolation conditions (150 mM NaCl) without crosslinking. Co-purified proteins were identified by mass spectrometry (MS) (Fig. 3c). Although there is some noise due to unspecific RNA-protein (RNP) interactions, this analysis revealed a significant enrichment of PECAM-1 and γ-catenin (Supplementary 1), compared with a non-targeting control oligonucleotide. LASSIE binding to PECAM-1 was validated by crosslinking immunoprecipitation (CLIP) resulting in a significant enrichment of LASSIE co-immunoprecipitated with an anti-PECAM-1 antibody compared with isotype control (IgG) (Fig. 3d). Gene ontology analysis revealed LASSIE interacting proteins as components of EC junctions and the protein processing machinery in the endoplasmic reticulum (ER) (Supplementary 2). The subcellular distribution of LASSIE and the interaction with identified proteins were consequently analyzed by RNA in situ hybridization. A branched DNA (bDNA) technique^32^ was applied using 20 oligonucleotides distributed along the LASSIE transcript ensuring specificity and enhancement of the RNA signal. A distinct LASSIE signal was observed co-localizing with junction proteins VE-cadherin, PECAM-1, γ-catenin and the ER marker calreticulin (Fig. 3e, f), consistent with the data obtained from LASSIE-AS purification. Specificity of the LASSIE probes was confirmed in LASSIE-silenced HUVECs, displaying a significantly decreased RNA signal (Supplementary Fig. 4d, e). Taken into account that PECAM-1, γ-catenin, and VE-cadherin display a rather membrane-associated localization, the latter partly overlapping with the ER signal, these results indicate that most of the LASSIE transcript is located close to the ER or the cell plasma membrane.

### LASSIE regulates endothelial barrier integrity and the response to laminar shear stress

**Fig. 4:**
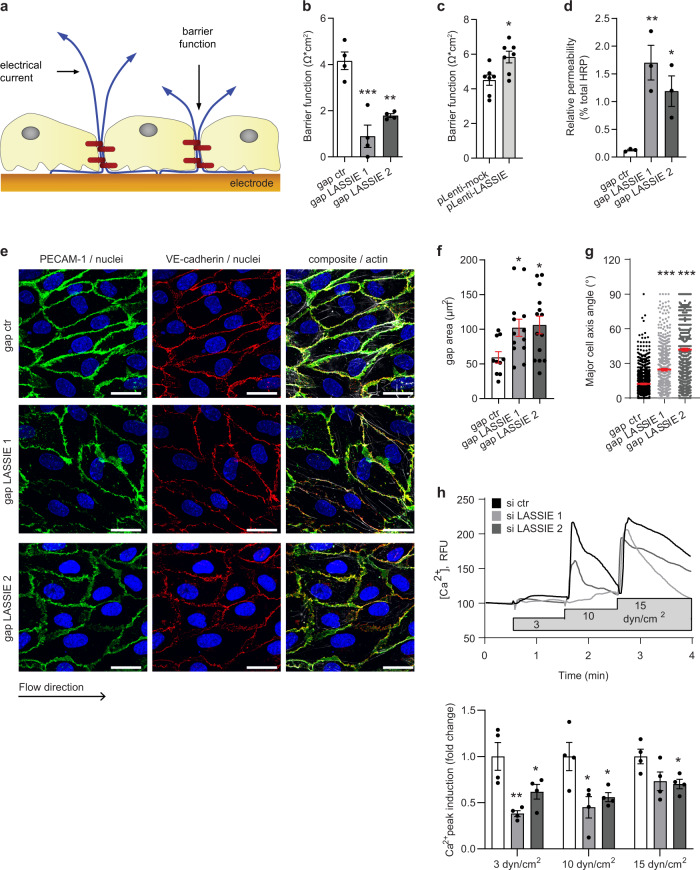
LASSIE regulates endothelial barrier integrity and the response to laminar shear stress. **a** Schematic depiction of the electrical cell-substrate impedance sensing (ECIS) technique to assess endothelial barrier function. **b**, **c** The effect of GapmeR-mediated (gap) silencing of LASSIE (*n* = 4; one-way ANOVA; compared with gap ctr: *p* = 0.0002 (gap LASSIE 1); *p* = 0.0023 (gap LASSIE 2)) and lentiviral-induced (Lenti) LASSIE overexpression (*n* = 7; unpaired *t*-test: *p* = 0.0121) on HUVECs barrier integrity was assessed by ECIS at 400 Hz. **d**–**g** HUVECs were treated with anti-LASSIE or control (ctr) GapmeR (gap). **d** Cells were seeded in Transwells and HRP passage through the endothelial monolayer was assessed by absorption measurements (450 nm) and shown as percentage of total HRP (*n* = 3; one-way ANOVA; compared with gap ctr: *p* = 0.007 (gap LASSIE 1); *p* = 0.0403 (gap LASSIE 2)). **e**–**g** Cells were exposed to laminar shear stress (20 dyn/cm^2^ for 48 h) and immunostained for VE-cadherin (red), PECAM-1 (green), and F-Actin (white). Nuclei were stained with DAPI (blue). Representative images are shown. Scale bars are 25 µm. **f** Quantification of the gap area between neighboring cells (*n* = 10 (gap ctr), *n* = 13 (gap LASSIE 1; *p* = 0.0425), *n* = 14 (gap LASSIE 2; *p* = 0.0226) over two independent experiments; one-way ANOVA (compared with gap ctr)). **g** Shear stress-induced cell alignment was quantified by determining the angle of the major cell axis (*n* = 1217 (gap ctr), *n* = 636 (gap LASSIE 1; *p* = 0 < 0.0001), *n* = 1159 (gap LASSIE 2; *p* < 0.0001)) over two independent experiments; one-way ANOVA (compared with gap ctr). **h** Fluo-4-loaded HUAECs were transfected with anti-LASSIE or control (ctr) siRNA (si) and exposed to indicated shear stress rates. Intracellular calcium [Ca^2+^]_I_ was determined as fluorescence intensity (RFU, relative fluorescence units). Representative graph is shown. Induction of [Ca^2+^]_I_ was quantified (*n* = 4; one-way ANOVA; compared with si ctr; 3 dyn: *p* = 0.0033 (si LASSIE 1); *p* = 0.0428 (si LASSIE 2); 10 dyn: *p* = 0.0141 (si LASSIE 1); *p* = 0.0412 (si LASSIE 2); 15 dyn: *p* = 0.0714 (si LASSIE 1); *p* = 0.0465 (si LASSIE 2)) (*p < 0.05; **p < 0.01; ***p < 0.001).

As LASSIE interacting proteins PECAM-1 and γ-catenin are part of endothelial junctional complexes and thus contribute to endothelial barrier function, we analyzed the influence of LASSIE in this process using the electric cell-substrate impedance sensing (ECIS) system^33^ (Fig. 4a). Strikingly, LASSIE knockdown significantly decreased (Fig. 4b), whereas its lentiviral-mediated overexpression (Supplementary Table 3, Supplementary Fig. 5a) increased resistance of the HUVEC monolayer (Fig. 4c). In accordance, endothelial barrier function analyzed by Transwell assays confirmed a decrease in HUVEC monolayer integrity after knockdown of LASSIE (Fig. 4d). This effect is independent of caspase-induced apoptosis, as treatment with the pan-caspase inhibitor Z-VAD-FMK did not prevent barrier disruption due to silencing of LASSIE (Supplementary Fig. 5b, c). However, the expression of junction proteins PECAM-1 and VE-cadherin was neither changed on RNA level nor on cell surface protein level (Supplementary Fig. 5d, e). Furthermore, the gap area between shear stress-exposed cells was increased after knockdown of LASSIE as quantified by immunostaining (Fig. 4e, f). Since AJs in general and PECAM-1 in particular are crucially involved in sensing shear stress^16,34^, we next determined whether LASSIE is required for proper endothelial shear stress response. Assessment of EC morphology revealed disturbed cell alignment in the direction of flow after silencing LASSIE (Fig. 4g). One of the earliest detectable responses to the onset of shear stress is the influx of calcium ions. We therefore measured calcium influx upon exposure to shear stress pulses of 3, 10, and 15 dyn/cm^2^ and detected a decrease in calcium influx after knockdown of LASSIE, as compared with control cells (Fig. 4h). These results indicate a detrimental effect due to LASSIE silencing on barrier function and shear stress sensing in ECs.

### LASSIE stabilizes adherens junctions by regulating the association to the cytoskeleton

**Fig. 5:**
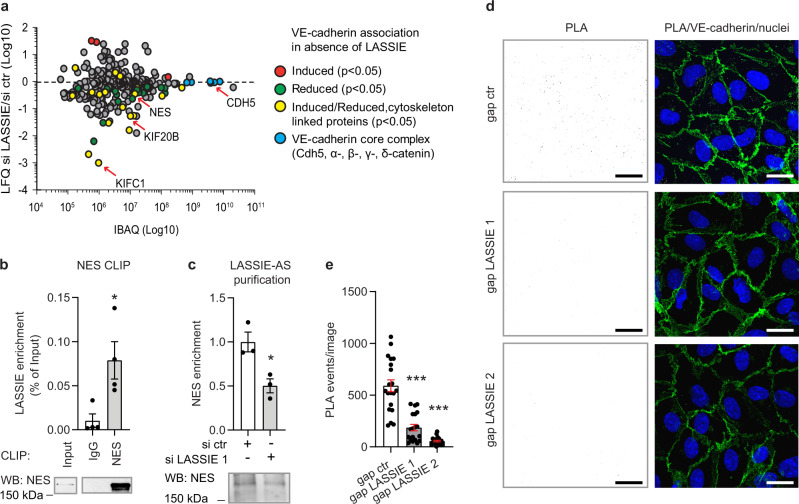
LASSIE stabilizes the association of the cytoskeleton to adherens junctions. **a** HUVEC lysates of anti-LASSIE or control (ctr) siRNA (si) treated cells were used for immunoprecipitation by incubation with an anti-VE-cadherin antibody. Captured proteins were analyzed by mass spectrometry. LFQ ratios of si LASSIE (*n* = 4) to si ctr (*n* = 5) are plotted against the respective IBAQ values. Proteins with significantly altered changes in VE-cadherin association are marked (red: higher association, green: lower association, yellow: associated cytoskeletal proteins). Proteins of the VE-cadherin core complex are depicted in blue. **b** HUVEC cell lysates were incubated with an anti-nestin (NES) antibody and isotype control (IgG) for crosslinking RNA immunoprecipitation (CLIP). Enrichment of co-purified LASSIE RNA was detected by qRT-PCR and shown as percentage of the input fraction (*n* = 4; unpaired *t-*test compared with IgG; *p* = 0.023). CLIP efficiency was analyzed by western blotting (WB), 1% of the lysate was used as a control and was detected separately. **c** Endogenous LASSIE-protein complexes were captured from lysates of si ctr and anti-LASSIE (si LASSIE 1) treated HUVECs using an LASSIE-AS oligonucleotide. RNA-antisense purifications were immunoblotted for nestin (*n* = 3; unpaired *t-*test; *p* = 0.022). **d**, **e** HUVECs were treated with anti-LASSIE or ctr GapmeR (gap). Protein interaction of VE-cadherin and nestin was analyzed by proximity ligation assay (PLA). Each PLA event (white) is indicative for protein interaction. Cells were immunostained for VE-cadherin (green), nuclei were stained with DAPI (blue). **d** Representative images are shown. Scale bars are 20 µm. **e** PLA events were counted per image (*n* = 20 (gap ctr), *n* = 19 (gap LASSIE 1; *p* < 0.0001), *n* = 21 (gap LASSIE 2; *p* < 0.0001) over three independent experiments; one-way ANOVA (compared with gap ctr)) (*p < 0.05; **p < 0.01; ***p < 0.001).

The direct interaction of LASSIE with AJ proteins as well as its impact on shear stress sensing led us to hypothesize a direct influence of LASSIE on the composition of endothelial junctional complexes. To address this hypothesis, LASSIE-silenced HUVECs were subjected to co-immunoprecipitation using an anti-VE-cadherin antibody (Supplementary Fig. 6a). Differentially associated proteins were identified by MS. This analysis revealed a significantly decreased association of cytoskeleton-linked proteins with VE-cadherin after silencing of LASSIE (Fig. 5a, Supplementary 3). Merging the earlier identified LASSIE interacting proteins with differentially VE-cadherin associated proteins resulted in an overlap of five proteins (Supplementary Fig. 6b). One of those is nestin, a type VI IF, which associates 2.4-fold less with VE-cadherin in the absence of LASSIE. Co-immunoprecipitation of VE-cadherin using an anti-nestin antibody was performed in the presence/absence of RNase A. Treatment with RNase A significantly decreased the interaction of nestin and VE-cadherin (Supplementary Fig. 6c), demonstrating an RNA-sensitive protein–protein interaction. More specifically, LASSIE binding to nestin was validated by CLIP resulting in a significant enrichment of LASSIE co-immunoprecipitated with an anti-nestin antibody compared with isotype control (IgG) (Fig. 5b). The specific enrichment of nestin by LASSIE-AS purification was confirmed in LASSIE-silenced cells, as nestin enrichment was decreased in the absence of LASSIE, demonstrated by MS and western blotting (Fig. 5c, Supplementary Fig. 6d, Supplementary 4). Decreased interaction of VE-cadherin and nestin after knockdown of LASSIE was confirmed by proximity ligation assay (PLA) where the PLA signal is a measure of protein–protein interactions (Fig. 5d, e). We therefore hypothesized that the association of nestin IF to AJs is controlled by LASSIE. To test this hypothesis, we analyzed the distribution of nestin in LASSIE-silenced HUVECs by immunofluorescence. The nestin network appeared more contracted and was less connected to the cell membrane after silencing of LASSIE in comparison to control cells, as quantified by co-localization analysis (Supplementary Fig. 6e, f).

**Fig. 6:**
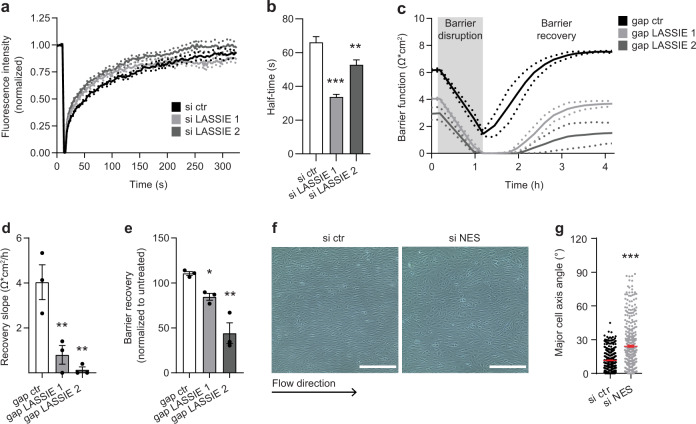
LASSIE regulates barrier function and shear stress sensing by stabilizing adherens junctions. **a**, **b** VE-cadherin-GFP overexpressing HUVECs were transfected with anti-LASSIE or ctr siRNA. Fluorescence recovery after photo bleaching (FRAP) was performed at junctional regions. **a** Fluorescence intensity is plotted against time. **b** Half-time was quantified as 50% of the respective plateau levels determined by nonlinear regression (*n* = 22 (si ctr), *n* = 26 (si LASSIE 1; *p* < 0.0001), *n* = 24 (si LASSIE 2; *p* = 0.0019)) over two independent experiments; one-way ANOVA (compared with si ctr). **c**–**e** HUVECs w**e**re treated with anti-LASSIE or ctr GapmeR and stimulated with microtubules destabilizing Nocodazole (350 nM) at 72 h post transfection. Barrier integrity was assessed by ECIS at 400 Hz. **d** The recovery slope was determined for a period of 1 h post stimulation (*n* = 3; one-way ANOVA; compared with gap ctr: *p* = 0.0044 (gap LASSIE 1); *p* = 0.0035 (gap LASSIE 2)). **e** Barrier recovery was analyzed by normalizing Nocodazole stimulated to untreated condition, 4 h post stimulation (*n* = 3; one-way ANOVA; compared with gap ctr: *p* = 0.0426 (gap LASSIE 1); *p* = 0.0012 (gap LASSIE 2)). **f**, **g** HUVECs were treated with anti-nestin (NES) or ctr siRNA and exposed to laminar shear stress (20 dyn/cm^2^ for 48 h). **f** Representative bright field images are shown. Scale bars are 400 µm. **g** Shear stress-induced cell alignment was quantified by determining the angle of the major cell axis (*n* = 414 (si ctr), *n* = 412 (si NES) over two independent experiments; unpaired *t*-test: *p* < 0.0001) (*p < 0.05; **p < 0.01; ***p < 0.001).

Next, we analyzed fluorescence recovery after photo bleaching (FRAP) of LASSIE-silenced HUVECs expressing GFP-labeled VE-cadherin, with the hypothesis that LASSIE-controlled cytoskeleton association affects VE-cadherin mobility. This assay revealed a faster recovery of the bleached linear junctional area (Fig. 6a, b), suggesting a higher mobility of VE-cadherin in LASSIE-silenced cells, which is indicative of decreased association with the cytoskeleton.

To assess the involvement of the cytoskeleton and the role of LASSIE in shear stress sensing and barrier function, we used pharmacological inhibitors to disrupt the cytoskeleton. Since there are no inhibitors to specifically disrupt IFs, we used Nocodazole, which effectively disrupts both the MT and IF networks^35^, and analyzed the effect on barrier function. Nocodazole treatment drastically decreased endothelial barrier integrity, as previously described^36^. The barrier was recovered within 4 h and even improved compared with before Nocodazole treatment. However, barrier recovery after Nocodazole treatment was incomplete in the absence of LASSIE (Fig. 6c–e). LASSIE knockdown did not affect recovery of the MT network per se, as analyzed by immunofluorescence (Supplementary Fig. 7), indicating that rather the interaction between the MT cytoskeleton and the barrier function-controlling cell–cell junctions is regulated by LASSIE. To more specifically address the role of the IF network in the LASSIE-controlled regulation of shear stress sensing and barrier function, we next used siRNA-mediated silencing of the IF protein nestin (Supplementary Fig. 8a–c). Loss of nestin did not reduce endothelial barrier function (Supplementary Fig. 8d), indicating that LASSIE-mediated barrier regulation is controlled by the MT cytoskeleton. However, silencing of nestin resulted in loss of cell alignment in response to flow (Fig. 6f, g), similar to LASSIE loss-of-function. These results propose a crucial role of LASSIE in connecting the nestin IF system to AJs and thereby stabilizing AJs. This connection and the presence of LASSIE and nestin are essential for normal shear stress sensing in ECs.

## Discussion

This study identifies the shear stress-induced lncRNA LASSIE as a crucial regulator of shear stress sensing and barrier function in ECs. Its association with protein complexes that are involved in endothelial junctions like PECAM-1 and γ-catenin as well as IF protein nestin, together with loss of interaction between VE-cadherin and cytoskeleton-associated proteins, indicate a crucial role of LASSIE in the cytoskeletal association to endothelial junctions. Indeed, silencing of LASSIE affected the stabilization of AJs by the cytoskeleton and thereby barrier function and shear stress responsiveness. Our results point out the importance of intact AJ stabilization through the IF cytoskeleton for normal EC function, mediated by the lncRNA LASSIE (Supplementary Fig. 8g).

Like many shear stress-induced genes, LASSIE expression is KLF2-dependent (Fig. 1b, c). KLF2 is a well-described transcription factor that is responsible for atheroprotective gene transcription in response to LSS^1–3^. Knockdown of KLF2 in HUVECs exposed to LSS did not completely abolish LASSIE expression (Fig. 1c), we therefore cannot exclude other transcription factors like KLF4 to be involved in the transcriptional regulation of LASSIE. Other lncRNAs are described to display shear stress-dependent expression^37,38^. Most lncRNAs are poorly evolutionary conserved on sequence level and are hypothesized to display locus or secondary structure conservation^39^. In this study, we identified a locus-conserved zebrafish homologue of LASSIE. Although shear stress-dependent expression of LASSIE (Fig. 1d, e) indicates functional conservation between human and zebrafish, further studies are required.

We identified the IF protein nestin as well as the AJ components PECAM-1 and γ-catenin as LASSIE interaction partners (Figs. 3c, 5b, Supplementary 1). Little has been described about the function of nestin in ECs. However, PECAM-1 is described to act as a shear stress sensor through association with vimentin IF, most likely via γ-catenin^14,16^. In this context, silencing of vimentin has been previously described to reduce the mechanical resistance to flow in immortalized human bone marrow ECs^40^. Furthermore, vimentin is localized in parallel to MT in ECs^41^ and reported to regulate barrier function in vivo^42^ and in cultured rat pulmonary microvascular ECs^43^. In HUVECs, silencing of vimentin alone did not affect the cell alignment in the direction of flow, likely due to compensation of other IF proteins (Supplementary Fig. 8e, f). In contrast, silencing of either LASSIE or nestin did drastically disturb the cell alignment in the direction of flow (Figs. 4g, 6f, g). This study describes the IF protein nestin to be associated with EC junctions and to be involved in shear stress sensing. The interaction of VE-cadherin and nestin is dependent on RNAs, as VE-cadherin co-immunoprecipitation with a nestin antibody was decreased in the presence of RNase A (Supplementary Fig. 6c). More specifically, the absence of LASSIE induces the dissociation of the VE-cadherin-nestin complex (Fig. 5a, d, e), suggesting that LASSIE mediates the attachment of IF protein nestin to AJs, essential for normal shear stress sensing.

Our results suggest that LASSIE is a crucial component of EC-cell junction regulation by stabilizing AJs through IF and MT networks. Interaction of both cytoskeletal components with VE-cadherin was decreased in the absence of LASSIE, as shown by MS of VE-cadherin immunoprecipitates (Fig. 5a). Furthermore, FRAP of GFP-labeled VE-cadherin was enhanced in LASSIE-silenced HUVECs due to increased VE-cadherin mobility (Fig. 6a, b). Strikingly, nestin and vimentin seem not to play an essential role in endothelial barrier resistance (Supplementary Fig. 8d). This suggests that LASSIE-induced barrier stabilization is rather mediated through the interaction of the MT cytoskeleton and the barrier function-controlling cell–cell junctions, as barrier recovery after Nocodazole treatment was incomplete in the absence of LASSIE (Fig. 6c–e). Crosstalk mechanisms between IF and MT have been described before^35,44^. Compensation by the MT network might thus account for the mild effect on barrier function induced by silencing of IFs. A clear distinction between a MT- and IF-mediated mechanism is consequently challenging and it cannot be excluded that the dissociation of either one of the cytoskeletal components might be a secondary effect due to crosstalk mechanisms. In short, this study indicates that LASSIE regulates barrier function by directly affecting the composition of junctional/cytoskeletal complexes.

In ECs, the lncRNA SENCR has recently been described to regulate VE-cadherin internalization through interaction with CKAP4^38^. Interestingly, in our study CKAP4 was purified with an anti-LASSIE oligonucleotide (Supplementary 1), likely an indirect interaction. However, we did not observe changes in the interaction between VE-cadherin and CKAP4 (Supplementary 3) and LASSIE does not regulate VE-cadherin internalization (Supplementary Fig. 5e), indicating distinct mechanisms by which these lncRNAs regulate barrier function.

Functional endothelial junctions are indispensable for cell homeostasis and junctional defects lead to cell apoptosis^45^. Accordingly, cell survival was impaired in LASSIE-silenced HUVECs, likely evoked by impaired cell–cell interactions, ultimately leading to impaired angiogenic sprouting (Fig. 2). Similar effects were observed after silencing VE-cadherin in HUVECs resulting in a decrease in continuous sprout formation (data not published). The VE-cadherin complex can indirectly regulate transcription as its binding partners β-, γ-, and p120-catenin can translocate to the nucleus for downstream transcriptional regulation, as described to be mediated by the lncRNA CYTOR^46^. Although our results indicate an influence of LASSIE on AJs, silencing of LASSIE does not influence the composition of the VE-cadherin-catenin complex (Fig. 5a, Supplementary 3). In accordance, LASSIE does not greatly affect global gene expression (Supplementary Fig. 4a).

LASSIE localizes close to the ER and co-localizes with junctional proteins PECAM-1, VE-cadherin, and γ-catenin (Fig. 3e, f). Bioinformatics pathway analysis of LASSIE interacting proteins indicate a possible role of LASSIE in protein processing in the ER and vesicle mediated transport to the plasma membrane (Supplementary 2, Supplementary Fig. 8g). This study thus suggests that LASSIE regulates protein domain assembly between junction proteins and nestin at the ER. Of note, LASSIE silencing does not affect PECAM-1 or VE-cadherin protein levels or cell membrane localization (Supplementary Fig. 5d, e). We therefore concluded that this complex is transported to the plasma membrane in a functionally incomplete state resulting in impaired endothelial barrier function and shear stress sensing in the absence of LASSIE. However, future studies are required to specify the impact of LASSIE on protein complex assembly at the ER.

Some studies already propose a cytoskeleton-associated role for lncRNAs^47^. Here we describe a lncRNA that functions as a direct link between the cytoskeleton and AJs, thereby regulating endothelial barrier and shear stress sensing.

In conclusion, this study provides evidence that the shear stress-regulated lncRNA LASSIE acts as a structural component of protein complexes. LASSIE is crucial for the cytoskeletal association to AJs and these data highlight LASSIE as a component in the regulation of endothelial barrier function and shear stress response.

## Materials and methods

### Cell culture

HUVECs and human umbilical artery endothelial cells (HUAECs) were purchased from Lonza and cultured in EC medium ECM (ScienCell) supplemented with 5% fetal calf serum (FCS, ScienCell), 1% penicillin/streptomycin (ScienCell) and endothelial growth supplement (ScienCell) or endothelial basal medium (EBM; Lonza) supplemented with EGM SingleQuots (Lonza), and 10% FCS (Invitrogen). Microvascular endothelial cells were isolated from pleura-free peripheral lung tissues and pulmonary artery endothelial cells from rings of the arteria pulmonalis, as described previously^48^ and cultured in ECM. Cells were used for experiments until passage four. Cell numbers were determined by an automated cell counter (Countess II FL, Invitrogen).

HEK293T cells were purchased from ATCC and cultured in Dulbecco’s modified Eagle medium (Lonza) supplemented with 10% FCS, 1% penicillin/streptomycin, 1% L-glutamin, and 1% pyruvate. Cells were used until passage 35 for production of lentiviral particles.

All cell types were cultured at 37 °C in a 5% CO_2_ atmosphere and tested negative for mycoplasma.

### Antibodies, oligonucleotides, and inhibitors

Information on antibodies and oligonucleotides used in this study is provided in Supplementary Tables 1–2. Primers, DNA oligonucleotides, and siRNAs were purchased from Sigma-Aldrich, LNA GapmeRs from Exiqon, and 2′O-Me-RNA probes from Integrated DNA Technologies. Protease and phosphatase inhibitors were purchased from Roche and RNase inhibitor from Thermo Scientific.

### Cell transfections and stimulations

HUVECs were transfected at 60–70% confluence with LNA GapmeRs or siRNAs using Lipofectamine RNAiMax (Thermo Scientific) according to the manufacturer’s protocol. Nocodazole (350 nM, Sigma-Aldrich) and Z-VAD-FMK (50 µM, Selleckchem) were diluted in the respective medium for stimulation of HUVECs.

### 5′ and 3′ RACE (rapid amplification of cDNA ends)

LncRNA transcript variants were identified by 5′ and 3′ RACE using the SMARTer™ RACE cDNA Amplification Kit (Clontech) according to the manufacturer’s protocol. Briefly, RNA of KLF2 overexpressing HUVECs was reverse transcribed using specific 5′ or 3′ end binding primers. Obtained cDNA was used as a template for 5′ and 3′ RACE PCRs with gene-specific primers. Gel-purified RACE PCR products were sequenced directly or cloned into pJET1.2/blunt vector (CloneJET PCR cloning kit, Thermo Scientific) according to the manufacturer’s protocol and sequenced subsequently. Sequencing was performed by Eurofins Genomics

### Lentiviral constructs

For lentiviral overexpression, the LASSIE transcript variant 1 sequence (Supplementary Table 3) was cloned into pLenti4/V5-DEST™ Gateway™ vector (Thermo Scientific). Lentiviral particles were generated by transfection of HEK293T cells with viral packaging plasmids pCMVΔR8.91, pMD2.G, and pLenti4/V5-DEST-LASSIE plasmid using GeneJuice^®^ Transfection Reagent (Merck Millipore). Empty pLenti4/V5-DEST™ was used as a control. Supernatants were collected 48 and 72 h after transfection and concentrated by centrifugation (690 × *g*, 4 °C) using Amicon Ultra-15 Centrifugal Filter Units (Merck Millipore). Long-term overexpression of VE-Cadherin-GFP^49^ or KLF2 as well as shRNA mediated silencing of KLF2^1^ was performed as previously described. HUVECs treated with lentivirus for LASSIE overexpression were washed with medium 24 h after virus transduction. Experiments were performed 72 h after virus transduction.

### RNA isolation and RT-qPCR

Total RNA from cultured HUVECs was isolated and DNase digested using the Direct-zol RNA miniprep kit (Zymo Research). 1 µg RNA was reverse transcribed using the iScript™ cDNA Synthesis Kit (Bio-rad). cDNA was diluted to a final concentration of 5 ng/µl (assuming a transcription efficiency of 100%) and used as a template for qPCR (iQ™ SYBR Green Supermix, Bio-rad) performed in a C1000 Touch™ Thermal Cycler (Bio-rad). mRNA expression levels of human RPLP0 and glyceraldehyde-3-phosphate dehydrogenase (GAPDH) were used for normalization and relative expression levels were subsequently calculated as values of 2^−^^ΔCt^.

### Nuclear and cytoplasmic cell fractionation

Nuclear and cytoplasmic fractions were separated as previously described^50^. Briefly, HUVECs were collected by cell scraping and centrifugation (5 min at 500 × *g*, 4 °C). Cell pellets were treated with cytoplasmic lysis buffer (10 mM Tris-HCl pH 7.5, 10 mM NaCl, 3 mM MgCl_2_, 0.5% Nonidet P-40 (NP-40)) and incubated on ice for 5 min. Cells were spun down and supernatants were collected as cytoplasmic fraction. Pelleted nuclei were washed with cytoplasmic lysis buffer and incubated with nucleic lysis buffer (10 mM Tris-HCl pH 7.5, 150 mM NaCl, 3 mM MgCl_2_) for 5 min on ice. Cytoplasmic fraction and pelleted nuclei were treated with Trizol and RNA was extracted from both fractions. Equal volumes of RNA were used for cDNA synthesis.

### Shear stress

In all, 8 × 10^4^ HUVECs were seeded in μ-slides I^0.4^ (Ibidi) and allowed to attach for 6 h. Cells were exposed to laminar (20 dyn/cm^2^ for 72 h) or oscillatory shear stress (20 dyn/cm^2^ for 14 h), controlled by the Ibidi perfusion system. The same cell number was seeded per well of a 24-well plate as static control. GapmeR/siRNA-treated cells were seeded in shear slides 20 h after transfection and were exposed to LSS for 48 h.

Shear stress analyses of bright field images were conducted with the straight line tool in ImageJ. Major and minor axes were measured for calculation of the length ratio. For analysis of the cell angle, cells with a length ratio smaller than 1 were excluded. The angle of the major axis of more than 600 cells per condition was compared from two independent experiments. Fluorescence images were used to quantify the gap area between cells by measuring the cell free area in ImageJ.

Intracellular calcium concentration [Ca^2+^]_I_ in response to shear stress was determined as described previously^51^. Briefly, HUAECs were seeded in a microfluidic plate (Fluxion) and loaded with Fluo-4 AM (Molecular Probes). Cells were exposed to LSS (3; 10 and 15 dyn/cm^2^) generated by the BioFlux 200 system (fluxion). [Ca^2+^]_I_ was determined as fluorescence intensity (RFU, relative units). [Ca^2+^]_I_ peak induction was quantified by setting individual baselines for each condition and each shear stress rate (RFU (peak)–RFU (baseline)).

### RNA sequencing

RNA sequencing data of HUVECs exposed to LSS was obtained from the NCBI GEO database (accession number GSE54384)^28^.

### Exon array

Global gene expression was analyzed by Affymetrix Human Exon 1.0 ST Array. Briefly, RNA was isolated from HUVECs treated with control or anti-LASSIE GapmeR for 48 h as described above. cDNA synthesis, labeling, hybridization, and scanning was performed by Atlas Biolabs GmbH (Berlin). Microarray data were normalized by quantile normalization. The exon array data have been deposited in the NCBI GEO database under the accession number GSE146110.

### In vitro sprouting assay

EC function was studied in a spheroid sprouting assay, as previously described^52^. Briefly, GapmeR-treated HUVECs (24 h post transfection) were seeded in ECM containing methylcellulose (20%) into a non-adherent round-bottom 96-well plate to allow one spheroid to be formed per well. Spheroids were collected after 24 h and embedded into a collagen type I gel (BD Biosciences) in a 24-well plate. After polymerization (30 min), ECM (VEGF, 50 ng/ml) was added on top and the plate incubated for 24 h.

EC spheroids were analyzed by bright field microscopy (Axio Observer Z1.0 microscope, Zeiss, magnification: 10×) using the Axio Vision 4.8 analysis software (Carl Zeiss). Cumulative and discontinuous sprout length of each spheroid was measured. Subtraction of the cumulative sprout length from the maximal distance of the migrated cell was defined as discontinuous sprout length.

### Cell apoptosis assays

Cell apoptosis was assessed using the Apo-ONE^®^ Homogeneous Caspase-3/7 Assay (Promega) according the manufacturer’s protocol. HUVECs were treated with pan-caspase inhibitor Z-VAD-FMK (50 µM in DMSO; Selleckchem) for 24 h and with Staurosporine (50 nM in DMSO; Sigma-Aldrich; final DMSO concentration 0.1%) for 4 h prior to measurement. Control cells were treated with 0.1% DMSO.

Binding of Annexin V to the cell surface and DNA intercalation of 7-AAD was analyzed by flow cytometry. Cells were trypsinized and spun down (5 min, 500 × *g*, 4 °C). Pellets were washed with 1× Annexin V binding buffer (BD Biosciences). 7-AAD (BD Biosciences) and the anti-Annexin V-V450 antibody (BD Biosciences) were added (1:50 in binding buffer) and incubated for 15 min at RT in the dark. Apoptosis was quantified by flow cytometry using a FACS Canto II device (BD Biosciences).

### Cell proliferation assays

Cell proliferation was assessed using the Click-iT^®^ EdU Microplate Assay (Invitrogen) according to the manufacturer’s protocol and by cell counting. Transfected HUVECs were counted at 24, 48, and 72 h using the Nucleocounter 2000 (ChemoMetec A/S).

### Flow cytometry

Cell surface expression of VE-Cadherin and PECAM-1 was analyzed in HUVECs by flow cytometry. Briefly, transfected HUVECs were detached with Accutase and washed in cold incubation buffer (0.1% BSA in PBS). Cells were blocked (5% BSA in PBS) for 30 min on ice and subsequently incubated with fluorophore-labeled antibodies for 30 min on ice. Protein cell surface expression was analyzed using a FACSCalibur™ device (BD Biosciences).

### Scratch wound healing assay

Cell migration chambers (Ibidi) were placed in a 24-well tissue culture dish. Cells were seeded into each half-chamber and grown overnight. After removal of the inserts, lateral cell migration was visualized by bright field microscopy (Axio Observer Z1.0 microscope, Zeiss, magnification: 10×). Pictures were taken at 0, 3, 6, and 8 h after removal of the inserts. Quantitative assay analysis was performed in ImageJ. The area covered by cells was determined for the indicated time points.

### Endothelial integrity measurement

Endothelial barrier function was measured by the ECIS system (Applied BioPhysics) as described previously^53^. Briefly, 40,000 HUVECs (24 h post transfection) were seeded per well of a gelatin-coated (1%) 96W1E + PET plate (Applied BioPhysics). The pan-caspase inhibitor Z-VAD-FMK (50 µM in DMSO; Selleckchem; final DMSO concentration 0.1%) was added 4 h post transfection and during seeding of transfected cells in the ECIS plate. Control cells were treated with 0.1% DMSO. Endothelial barrier integrity was analyzed after 48 h when cells formed a stable monolayer. Barrier resistance (*R*_*b*_) was measured by applying an alternating current of 400 Hz resulting in a potential which is detected by the ECIS instrument Zθ (Applied BioPhysics), impedance is determined according to Ohm’s law. In all, 100,000 HUVECs were seeded in an 8W1E ECIS array (Applied Biophysics) for analyzing cell migration by inducing cell wounding through lethal electroporation. Wound repair was observed over a period of 4 h, the area under the impedance curve (4000 Hz) was calculated.

### Transwell assay

Endothelial barrier integrity was analyzed by HRP passage through the endothelial monolayer. Overall, 100,000 HUVECs (24 h post transfection) were seeded into gelatin-coated (1%) ThinCerts™ cell culture inserts (pore sixe: 3.0 mm, Greiner Bio-one). Forty-eight hours post transfection, 5 mg/ml HRP (Sigma-Aldrich) was added to the upper compartment for 1 h. HRP passage was determined by absorption measurements (450 nm) of the lower and upper compartment after adding TetraMethylBenzidine (Merck Millipore) and sulfuric acid.

### RNase H accessibility assay

Accessibility of LASSIE RNA was assessed as previously described^27^. HUVECs were lysed (50 mM Tris-HCl pH 8, 150 mM NaCl, 0.5% NP-40, protease inhibitor) for 30 min on ice. Cell lysates were cleared by centrifugation (10 min, 16,000 × *g*, 4 °C) and supernatants were adjusted to a volume of 1 ml (50 mM Tris-HCl pH 8, 60 mM NaCl, 75 mM KCl, 3 mM MgCl_2_, 10 mM DTT, 40 U RNase inhibitor). 100 pmol of DNA LASSIE 1 oligonucleotide was incubated with 100 µl cell lysate for 2 h at 4 °C under continuous rotation. 2.5 U RNase H (New England Biolabs) was added and incubated for 20 min at 37 °C (350 rpm). Finally, Trizol was added and RNA accessibility was analyzed by RT-qPCR.

### RNA-antisense purification

RNP complexes were captured as previously described^27^. In brief, HUVECs (one confluent 15 cm dish/condition) were lysed (50 mM Tris-HCl pH 8, 150 mM NaCl, 1 mM EDTA, 0.5% NP-40, protease inhibitor) for 30 min on ice. Cell lysates were cleared by centrifugation (10 min, 16,000 × *g*, 4 °C), adjusted to a volume of 1 ml (50 mM Tris-HCl pH 8, 150 mM NaCl, 75 mM KCl, 3 mM MgCl_2_, 10 mM DTT, 80 U RNase inhibitor) and pre-cleared for 2 h at 4 °C with 50 µl pre-blocked (glycogen and yeast tRNA (0.2 mg/ml, Sigma-Aldrich) for 2 h at 4 °C) streptavidin C1 beads (Thermo Scientific). Equal amount of protein lysates were used for siRNA-treated cells. RNP complexes were isolated by incubating lysates with 100 pmol desthiobiotin-coupled 2′O-Me-RNA anti-LASSIE and non-targeting control oligonucleotides for 1 h at 37 °C. RNP-oligonucleotide complexes were captured using 100 μl pre-blocked beads for 1 h at 37 °C. Beads were washed four times (lysis buffer containing 0.05% NP-40) followed by Biotin (100 μM) elution for 1 h at RT. Eluates were analyzed by RT-qPCR and MS. For MS analysis and protein enrichment analysis by western blotting of siRNA-treated cells, beads were washed twice (20 mM Tris-HCl pH 7.5, 10 mM NaCl, 1 mM EDTA, 0.05% NP-40) and once with the same buffer not containing NP-40 following RNA purification. Samples for MS were treated as described above. For protein enrichment analysis by western blotting, beads were treated with 5× sample buffer (312.5 mM Tris pH 6.8, 50% glycerol, 0.37 mM Bromphenol blue, 347 mM SDS, 2.5% β-mercaptoethanol).

### Crosslinking RNA immunoprecipitation (CLIP)

HUVECs (one confluent 15 cm dish/condition) were UV-crosslinked (2 × 50 mJ/cm^2^, Analytic Jena) and lysed (PECAM-1 CLIP: 50 mM Tris pH 7.5, 150 mM NaCl, 1 mM EDTA, 0.5% Triton X-100, 0.5% Deoxycholate, 1 mM Na_2_VO_4_, 2 mM PMSF, protease inhibitor, 80 U RNase inhibitor; NES CLIP: 50 mM Tris pH 7.5, 150 mM NaCl, 1 mM EDTA, 0.5% NP-40, protease inhibitor) for 30 min on ice. Cell lysates were cleared by centrifugation (10 min, 16,000 × *g*, 4 °C) and supernatants were adjusted to a volume of 1 ml (50 mM Tris, pH 7.5, 150 mM NaCl, 1 mM EDTA, 1 mM DTT). For immunoprecipitation, 1 ml cell lysate was incubated with anti-PECAM-1, anti-nestin antibody or control IgG overnight at 4 °C. RNP complexes were captured with 50 µl Dynabeads protein G (Thermo Scientific) by incubation at 4 °C for 4 h. Beads were washed three times (20 mM Tris pH 7.5, 10 mM NaCl, 1 mM EDTA) and proteinase K (New England Biolabs) digested (200 mM Tris pH 8, 300 mM NaCl, 2% SDS, 25 mM EDTA, 6.4 U proteinase K) for 30 min at 55 °C. RNA was recovered by phenol/chloroform/isoamyl extraction and RNP binding was quantified by RT-qPCR.

### Immunoprecipitation

HUVECs (48 h post transfection) were lysed (25 mM Tris-HCl pH 7.5, 100 mM NaCl, 10 mM MgCl_2_, 1 mM EDTA, 10% glycerol, 1% NP-40, protease and phosphatase inhibitors) for 15 min on ice. For immunoprecipitation, 0.7 mg cell lysate was incubated with anti-VE-Cadherin antibody and 50 µl Dynabeads protein G (Thermo Scientific) for 1.5 h at 4 °C. Beads were washed with lysis and wash buffer (25 mM Tris-HCl pH 7.5, 100 mM NaCl, 10 mM MgCl_2_) and finally analyzed by MS. Proteins with a sequence coverage higher than 4% were used for further quantification.

For co-immunoprecipitation of VE-cadherin using an anti-nestin antibody, 100 µg/mL RNase A (Thermo Scientific) was added to the lysis buffer and the IP reaction to analyze the involvement of RNAs on the studied interaction.

### Mass spectrometry

RNA pulldown eluates and immunoprecipitation beads were analyzed by liquid chromatography/MS using a Q Exactive Plus (Thermo Scientific) mass spectrometer equipped with an ultra-high performance liquid chromatography unit (Dionex Ultimate 3000, Thermo Scientific) and a Nanospray Flex Ion-Source (Thermo Scientific). Data analysis was performed in MaxQuant 1.5.3.30^54^, Perseus 1.5.6.0^55^, and Excel (Microsoft Office 2013). The MS proteomics data have been deposited to the ProteomeXchange Consortium via the PRIDE^56^ partner repository with the dataset identifier PXD018724 (LASSIE-AS purification, Fig. 3c), PXD018734 (LASSIE-RNA purification, Supplementary Fig. 6d), PXD018725 (VE-Cadherin IP, Fig. 5a).

### Immunofluorescence

HUVECs were grown on gelatin (1%) coated coverslips at static condition or exposed to LSS. Cells were fixed in paraformaldehyde (3.7%) for 10 min at RT, permeabilized in Triton X-100 (0.1%) for 10 min at RT, blocked in BSA (2%) for 1 h at RT, and incubated with primary antibodies (in 2% BSA) overnight at 4 °C. Cells were stained with fluorophore conjugated secondary antibodies (Life technologies) and Acti-stain 555 phalloidin (Cytoskeleton) for 1 h in the dark. Nuclei were labeled with DAPI (Invitrogen). All washing steps were performed in HBSS with calcium and magnesium (Gibco), which was used for reagent dilution. Cells were imaged by confocal microscopy (Nikon A1R) using a 63 × 1.4 NA oil-immersion objective.

FRAP bleaching experiments were performed in HUVECs, virally transduced with VE-cadherin-GFP^49^. Cells were seeded in Lap-Tek (Thermo Scientfic) chamber slides and FRAP experiments were performed 48 h later with 488 nm laser illumination. Fluorescence recovery was measured by time-lapse imaging (63 × 1.4 NA oil-immersion objective, Leica SP8) using 50 iterations after photo bleaching. Fluorescence values were normalized to total fluorescence. Fluorescence recovery half-time was time was quantified as 50% of the respective plateau levels determined by nonlinear regression.

### Fluorescence in situ hybridization

Subcellular localization of LASSIE was assessed in HUVECs exposed to LSS using the ViewRNA ISH Cell Assay Kit (Thermo Scientific) according to the manufacturer’s instructions. Briefly, cells were fixed, permeabilized, blocked, and incubated with primary antibodies followed by fluorophore conjugated secondary antibody incubation. Hybridization of a Type 1 LASSIE-specific ViewRNA probe set (targeting LASSIE transcript variant 1) was performed at 40 °C for 3 h. The signal was increased in a sequential bDNA amplification step. bDNA structures were detected by Alexa Fluor 546 dyes. A Type 6 probe set targeting b-Actin was used as a positive control, detected by Alexa Fluor 647. Nuclei were labeled with DAPI (Invitrogen) and finally analyzed by confocal microscopy (Nikon A1R) using a 63 × 1.4 oil-immersion objective.

Co-localization analysis of LASSIE with different proteins was conducted in ImageJ by creating a mask of the LASSIE and the respective protein signal. Co-localization of the LASSIE signal was determined by overlaying both masks and counting the co-localizing particles.

### Proximity ligation assay

Protein–protein interactions were assessed using the Duolink^®^ PLA (Sigma-Aldrich) according to the manufacture’s protocol In brief, HUVECs were grown on gelatin-coated coverslips (1%), fixed in paraformaldehyde (3.7%) for 10 min at RT and permeabilized in Triton X-100 (0.1%) for 10 min at RT. Cells were blocked (1 h at 37 °C) and incubated with primary antibodies overnight (4 °C). Cells were incubated with the respective PLA probes for 1 h at 37 °C and subsequent ligation was performed for 30 min (37 °C). The amplification with polymerase was allowed for 100 min (37 °C) and cells were stained with an anti-VE-Cadherin antibody, nuclei were labeled with DAPI (Fluoromount-G with DAPI, Invitrogen). Protein–protein interactions were detected with 546 nm laser illumination by confocal microscopy (Nikon A1R) using a 63 × 1.4 oil-immersion objective. PLA events were counted in ImageJ.

### Western blot

HUVECs (48 h post transfection) were washed with ice cold PBS and lysed in RIPA Buffer (Sigma-Aldrich) supplemented with proteinase and phosphatase inhibitor (25 mM NaF, 10 mM Na_2_VO_4_, 10 mM Na_2_PO_7_, 5 mM β-glycerophosphate disodium salt hydrate) for 15 min on ice. Protein concentrations were determined by Bradford assays (Bio-rad) and lysates were treated with 5× sample buffer (312.5 mM Tris pH 6.8, 50% glycerol, 0.37 mM Bromphenol blue, 347 mM SDS, 2.5% β-mercaptoethanol). Equal protein amounts were separated by SDS-polyacrylamide gel electrophoresis and transferred to nitrocellulose membranes (GE Health care). Membranes were blocked in BSA (5% in TBS-T) for 1 h. Primary antibodies were diluted in blocking solution and incubated overnight (4 °C). GAPDH was used as a loading control. HRP-conjugated secondary anti-mouse/rabbit antibodies (Dako) were incubated for 1 h at RT. ECL detection (Merck Millipore) was used for visualization with the Amersham Imager 600 (GE Health Care). Band intensities were quantified in ImageQuant TL (GE Health Care).

### Morpholino studies in zebrafish

Zebrafish embryos were raised and maintained at 28.5 °C in E3 egg water. Fertilized fli1a:EGFP transgenic embryos were collected immediately after spawning. Morpholino oligonucleotides (Gene Tools) targeting tnnt2a and a control sequence (4 ng) were injected into one-cell stage fli1a:EGFP transgenic embryos^57^. Embryos were euthanized 72 hpf by MS-222, 20–30 embryos were collected for one sample dissociation. Embryos were dissociated by collagenase in HBSS at 28 °C for 15 min, reaction was stopped by adding three times volume of 10% FCS. Cells were passed through a 40 µm cell strainer into a FACS tube. Overall, 50,000 GFP positive cells were sorted as one sample for RNA isolation and qPCR analysis. Experiments were repeated three times independently. Expression of *klf2a* and *BC091967* was examined by RT-qPCR.

All zebrafish husbandries were performed under standard conditions, and all experiments were carried out in accordance with institutional (University of Cologne) and national ethical and animal welfare guidelines. All animal procedures conformed to EU Directive 86/609/EEC and Recommendation 2007/526/EC regarding the protection of animals used for experimental and other scientific purposes. The loss of function experiments using morpholinos was approved by the Landesamt Für Natur, Umwelt und Verbraucherschutz Nordrhein-Westfalen (LANUW NRW, Postfach 10 10 52, 45610 Recklinghausen) under the approval number 84-02.04.2014.A295.

### Statistics and reproducibility

GraphPad 7 (GraphPad Software) was used for statistical analyses. Comparison of two different conditions was analyzed by two-tailed Student’s *t* test or Mann–Whitney, multiple comparisons were performed by one-way or two-way ANOVA using Dunnett’s, Bonferroni, Holm’s–Sidak, Tukey’s, or Kruskal–Wallis correction. Data are expressed as means ± SEM, *p* < 0.05 was considered as statistically significant (*p < 0.05; **p < 0.01; ***p < 0.001). Outliers were identified by Grubbs method. The sample size *n* states the number of independent experiments, unless denoted otherwise. All results were reproduced in at least three technically independent replicates.

### Reporting summary

Further information on research design is available in the Nature Research Reporting Summary linked to this article.

## Supplementary information

Supplementary Information

Description of Additional Supplementary Files

Supplementary Data 1

Supplementary Data 2

Supplementary Data 3

Supplementary Data 4

Supplementary Data 5

Reporting Summary

Peer Review File

## Data Availability

All data generated and analyzed during this study are available from the corresponding author on reasonable request. Microarray data are deposited in the Gene Expression Omnibus repository under the accession number GSE146110. Proteomics data are deposited in the PRIDE archive under the accession numbers PXD018724, PXD018734, and PXD018725. Source data are provided as Supplementary Data 5.
