# Supplementary material for: Long non-coding RNA LASSIE regulates shear stress sensing and endothelial barrier function
Source: Commun Biol. 2020 May 26;3:265. doi: 10.1038/s42003-020-0987-0 (PMC7251106; doi:10.1038/s42003-020-0987-0)
Supplement: Supplementary file 1 — Supplementary Information [file 42003_2020_987_MOESM1_ESM.pdf]

Supplementary Figure 1

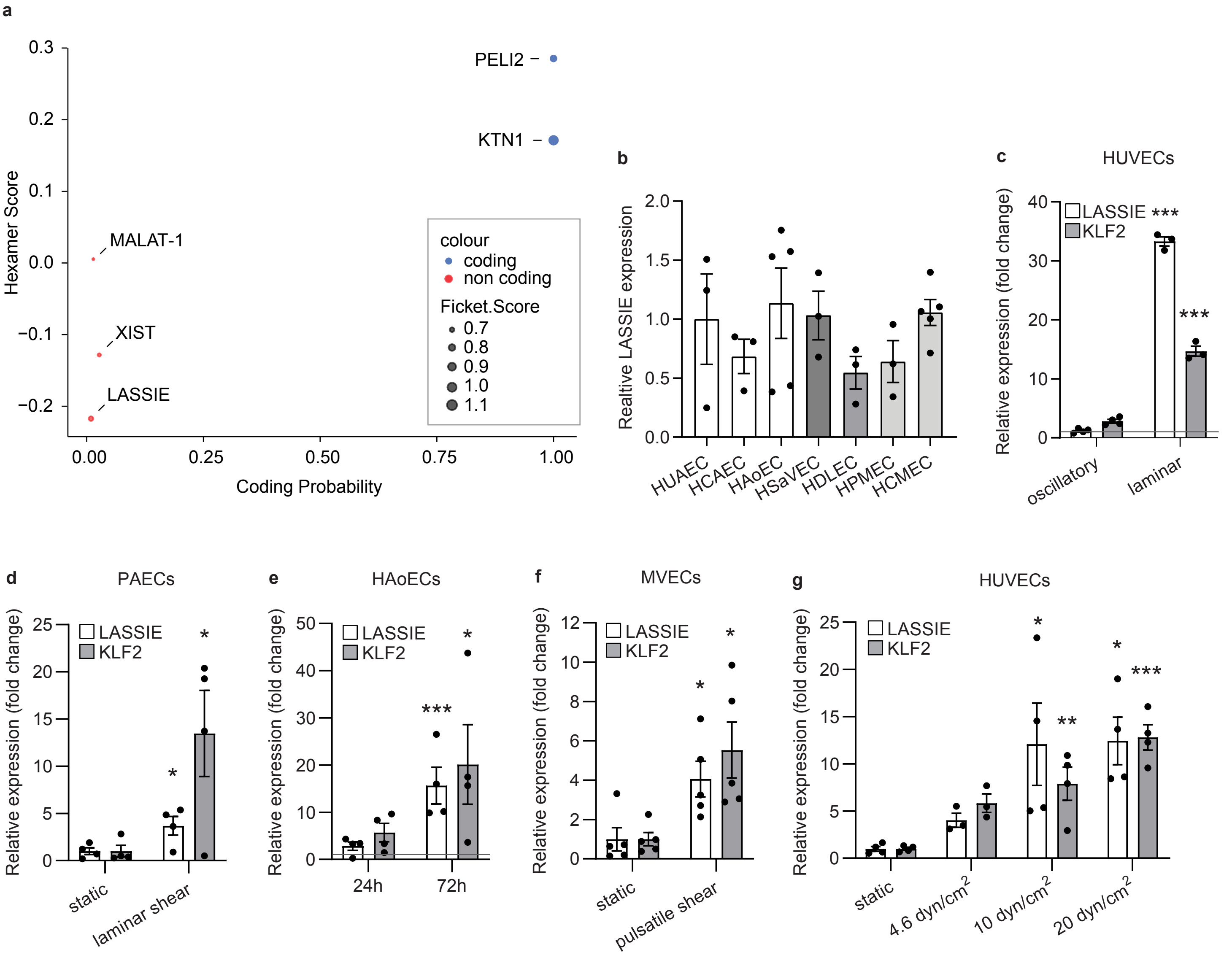

Supplementary Figure 1. (a) Coding potential of the lncRNAs MALAT-1, XIST and LASSIE were compared to those of protein coding genes PELI2 and KTN1 using the coding potential assessment tool CPAT (http://rna-cpat.sourceforge.net/). Hexamer usage bias (Hexamer Score) is plotted against the coding probability. **(b)** LASSIE expression in different EC types. RNA was isolated from different endothelial cell types, HUAEC (human umbilical artery ECs, n=3), HAoEC (human aortic ECs, n=3), HSaVEC (human saphenous vein ECs, n=5), HDLEC (human dermal lymphatic ECs, n=3), HPMEC (human pulmonary microvascular ECs, n=3), HCAEC (human coronary artery ECs, n=3) and HCMEC (human cardiac microvascular ECs, n=5). Expression of LASSIE was assessed by qRT-PCR (expression values were normalized to GAPDH mRNA). **(c-g)** Endothelial cells (ECs) were exposed to different types and magnitudes of shear stress for different time periods to analyze shear stress-dependent expression of LASSIE. Gene expression was assessed by qRT-PCR, KLF2 is shown as a shear stress-induced positive control. Expression is relative to respective static cells and normalized to housekeeping genes. **(c)** HUVECs were exposed to oscillatory (20 dyn/cm^2 for 14 h, n=4) and laminar shear stress (20 dyn/cm^2 for 72 h, n=3), normalized to GAPDH mRNA. Grey line depicts static control levels (2-way ANOVA; oscillatory vs. laminar shear stress; LASSIE: $p<0.0001$; KLF2 $p<0.0001$). **(d)** Pulmonary arterial ECs (PAECs) were exposed to laminar shear stress (15 dyn/cm^2) for 24 h, normalized to RPL27 mRNA (n=4; Unpaired t test; LASSIE: p=0.0444; KLF2: p=0.0351). **(e)** Human aortic ECs (HAoECs) were exposed to laminar shear stress (20 dyn/cm^2) for the indicated time periods, normalized to HPRT1 mRNA. Grey line depicts static control levels (n=4; 2-way ANOVA; LASSIE 72h: p=0.0005; KLF2 72h: p=0.0178). **(f)** Microvascular ECs (MVECs) were exposed to pulsatile shear stress (15 dyn/cm^2) for 24 h, normalized to RPLP0 mRNA (n=5; Unpaired t test; LASSIE: p=0.0226; KLF2: p=0.0148). **(g)** HUVECs were exposed to the indicates magnitudes of laminar shear stress for 24, normalized to GAPDH mRNA (n=4; 1-way ANOVA; static vs. 10 dyn/cm^2: LASSIE: p=0.0333; KLF2: p=0.0055; static vs. 20 dyn/cm^2: LASSIE: p=0.0283; KLF2: $p<0.0001$). (*$p<0.05$; **$p<0.01$; ***$p<0.001$)

Supplementary Figure 2

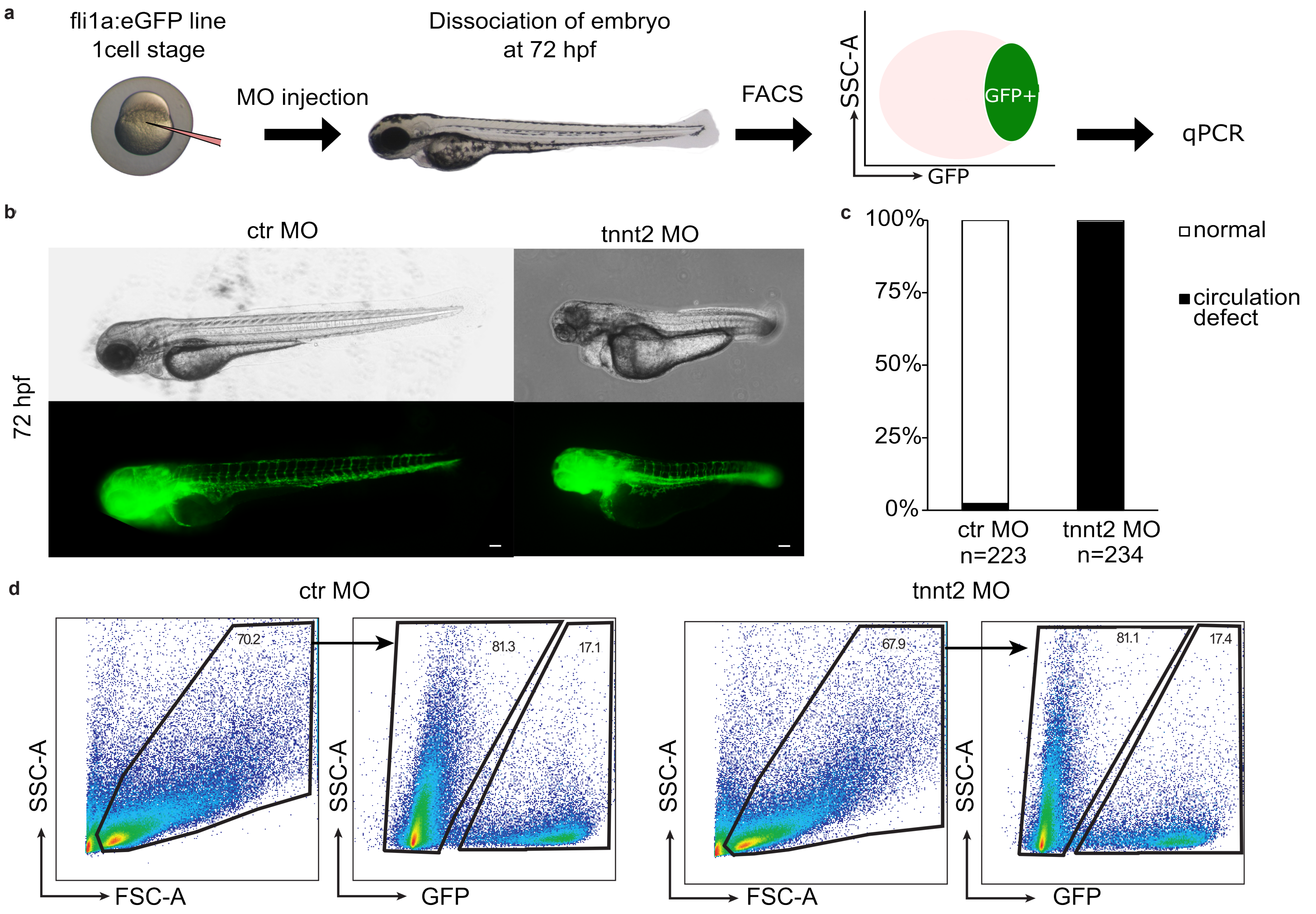

Supplementary Figure 2. (a-d) MO were injected into fli1a:eGFP one cell stage embryos. GFP positive cells of tnnt2a morphants (tnnt2a MO) relative to control morpholino treated zebrafish (ctr MO) were sorted by FACS and used for qRT-PCR to analyze shear stress-dependent expression of zebrafish LASSIE (BC091967). **(a)** Schematic representation of experimental strategy. **(b)** Representative lateral view of control MO and tnnt2 MO injected embryos at 72 hpf, scale bar are 100 µm. **(c)** Distribution of circulation defect embryos after MO treatment: ctr MO (n=223), tnnt2 MO (n=234) over three independent experiment. **(d)** Scatter plot of flow cytometry analysis and gating strategy for single cell sorting. (*p<0.05; **p<0.01; ***p<0.001).

Supplementary Figure 3

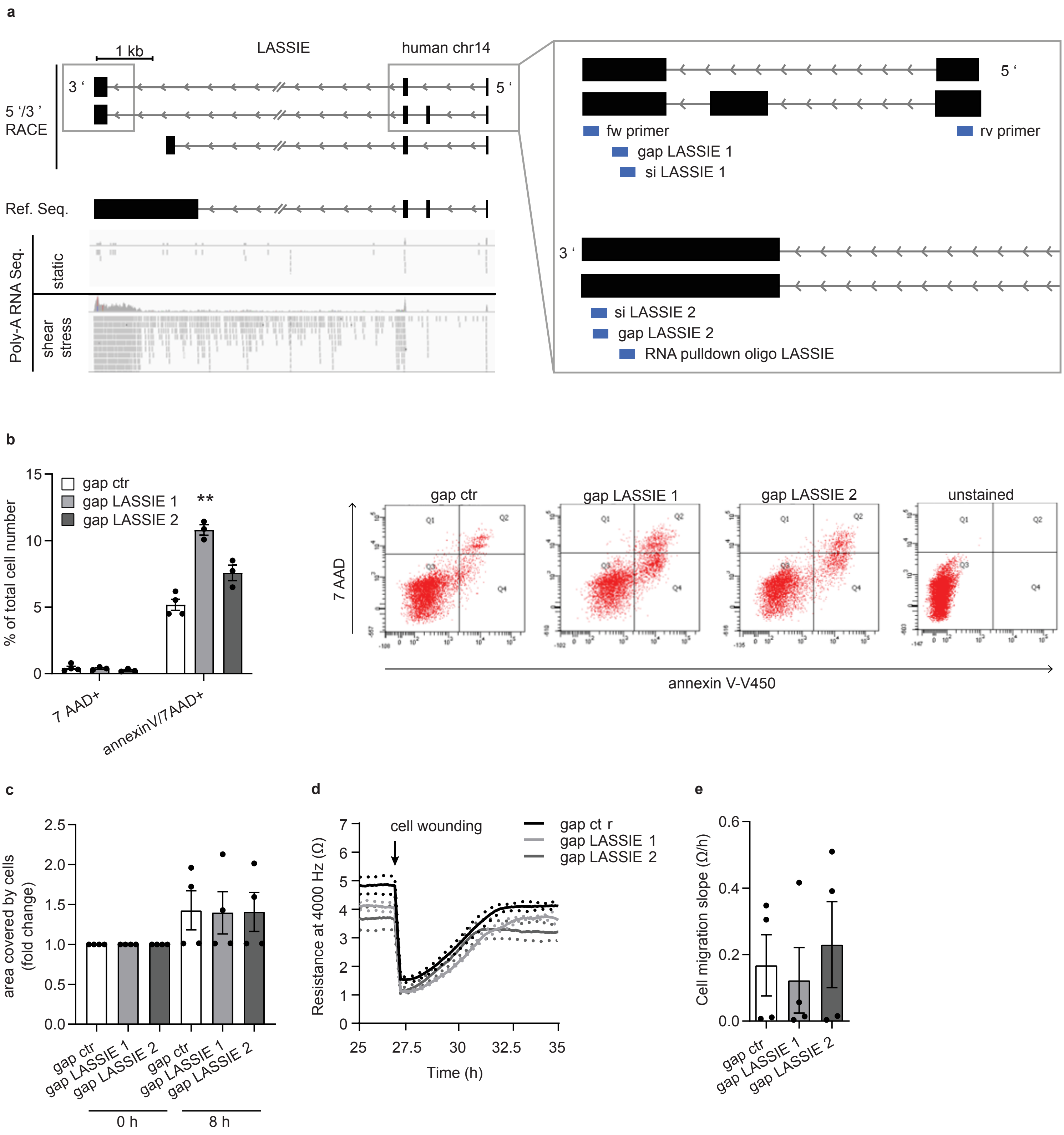

Supplementary Figure 3. (a) Structural depiction of the human LASSIE gene (not to scale). Depicted are three transcript variants of LASSIE identified by 5´ and 3´ RACE in HUVECs and the Ref.Seq. (NR_026796.1). Representative RNA sequencing reads of poly-adenylated RNA isolated from static and shear stress exposed HUVECs (20 dyn/cm^2 for 72 h) are shown. LASSIE-binding oligonucleotides used in this study are indicated in blue. **(b-e)** HUVECs were transfected with anti-LASSIE or control (ctr) GapmeR (gap), subsequent experiments were performed 48 h after transfection. **(b)** Apoptosis was assessed by flow cytometry of annexin V and 7-AAD stained cells (n=3; 1-way ANOVA; gap ctr 7aad/annexin vs. gap LASSIE 1 7aad/annexin: p=0.0069). **(c)** Cell migration was determined by scratch wound assay. The area covered by cells was quantified after 8 h and normalized to the start point (t=0 h), more than three areas were quantified per condition and experiment (n=4; 1-way ANOVA). **(d-e)** Migration was further assessed by Electrical Cell-Substrate Impedance Sensing (ECIS) measuring the resistance at 4000 Hz and wounding the cells at a stable monolayer situation (27 h). **(d)** One representative measurement with induced wounding at 27 h is shown. **(e)** Migration was quantified by calculating the recovery slope for 4 h post wounding (27-31 h; n=4; 1-way ANOVA). (*p<0.05; **p<0.01; ***p<0.001)

Supplementary Figure 4

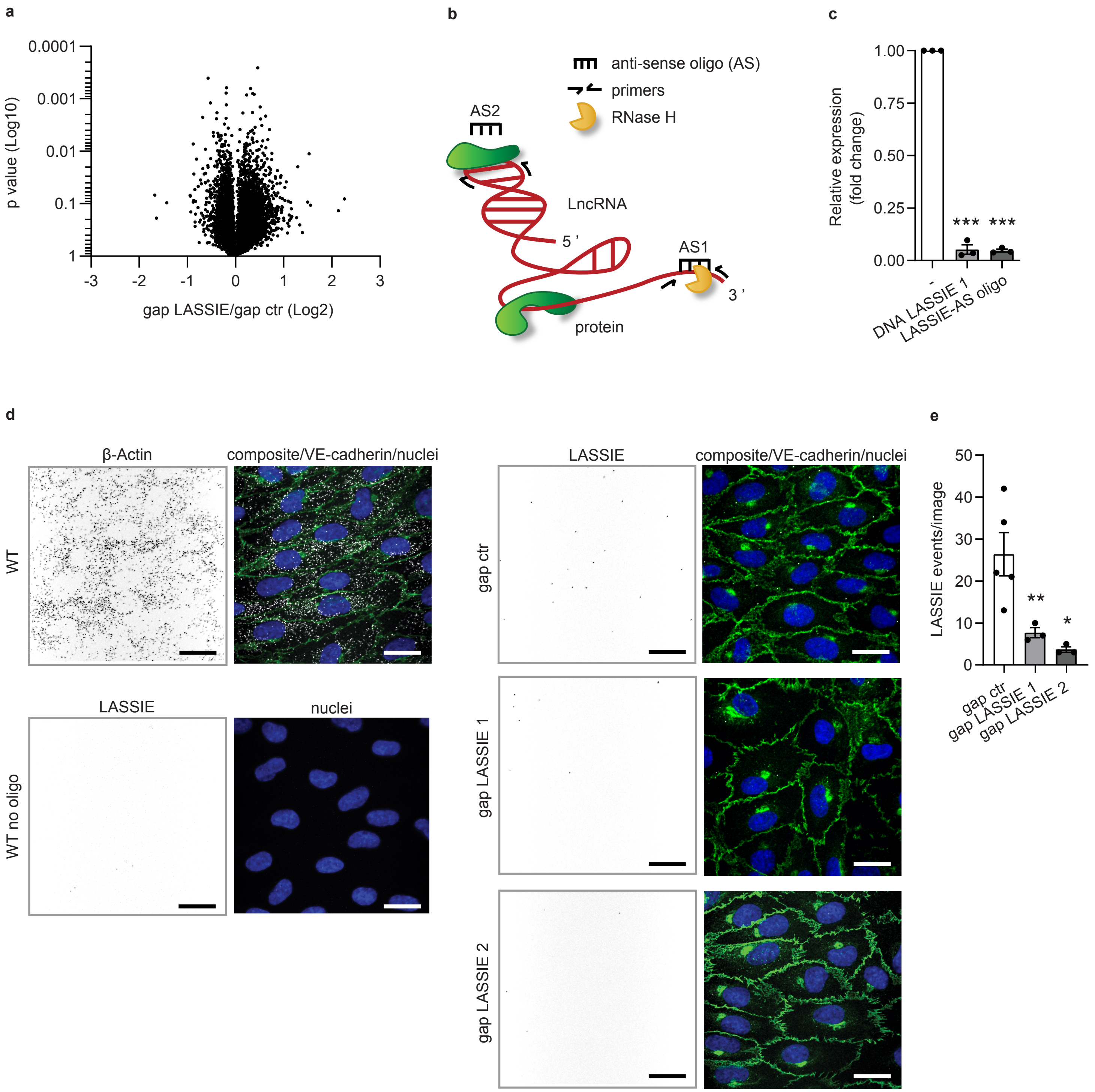

Supplementary Figure 4. (a) HUVECs were treated with anti-LASSIE or control (ctr) GapmeR (gap).The influence of LASSIE on global gene expression was assessed by exon array analysis (n=3). **(b)** Schematic depiction of lncRNA transcript accessibility test by RNase H cleavage. **(c)** HUVEC cell lysates were incubated with a LASSIE-antisense DNA oligo (LASSIE-AS oligo) or a positive control oligo (DNA LASSIE 1) which corresponds to the anti-LASSIE GapmeR sequence 1. Incubation with RNase H resulted in DNA-RNA heteroduplex degradation. This region could not be amplified using flanking primers in a subsequent qRT-PCR reaction, indicating binding of the tested oligo to the LASSIE transcript. Expression values were normalized to the control sample lacking oligo incubation (-), (n=3; 1-way ANOVA; - vs. DNA LASSIE 1: $p<0.0001$; - vs. LASSIE-AS oligo: $p<0.0001$). **(d-e)** Subcellular localization of LASSIE (white) was analyzed by ViewRNA® *in situ* hybridization in HUVECs exposed to laminar shear stress (20 dyn/cm^2 for 48 h). **(d)** *In situ* hybridization of β-Actin mRNA in wild type (WT) cells is shown as a positive control, cells without oligo incubation are shown as a negative control. LASSIE-AS probe specificity was analyzed in GapmeR anti-LASSIE treated cells. Cells were immunostained for VE-cadherin (green), nuclei were labelled with DAPI (blue). Representative images are shown. Scale bars are 20 µm. **(e)** GapmeR-mediated silencing of LASSIE was quantified; n=5 (gap ctr) n=3 (gap LASSIE 1) n=3 (gap LASSIE 2) in one representative experiment (1-way ANOVA; gap ctr vs. gap LASSIE 1: $p=0.0263$; gap LASSIE 2: $p=0.0100$). (*$p<0.05$; **$p<0.01$; ***$p<0.001$)

Supplementary Figure 5

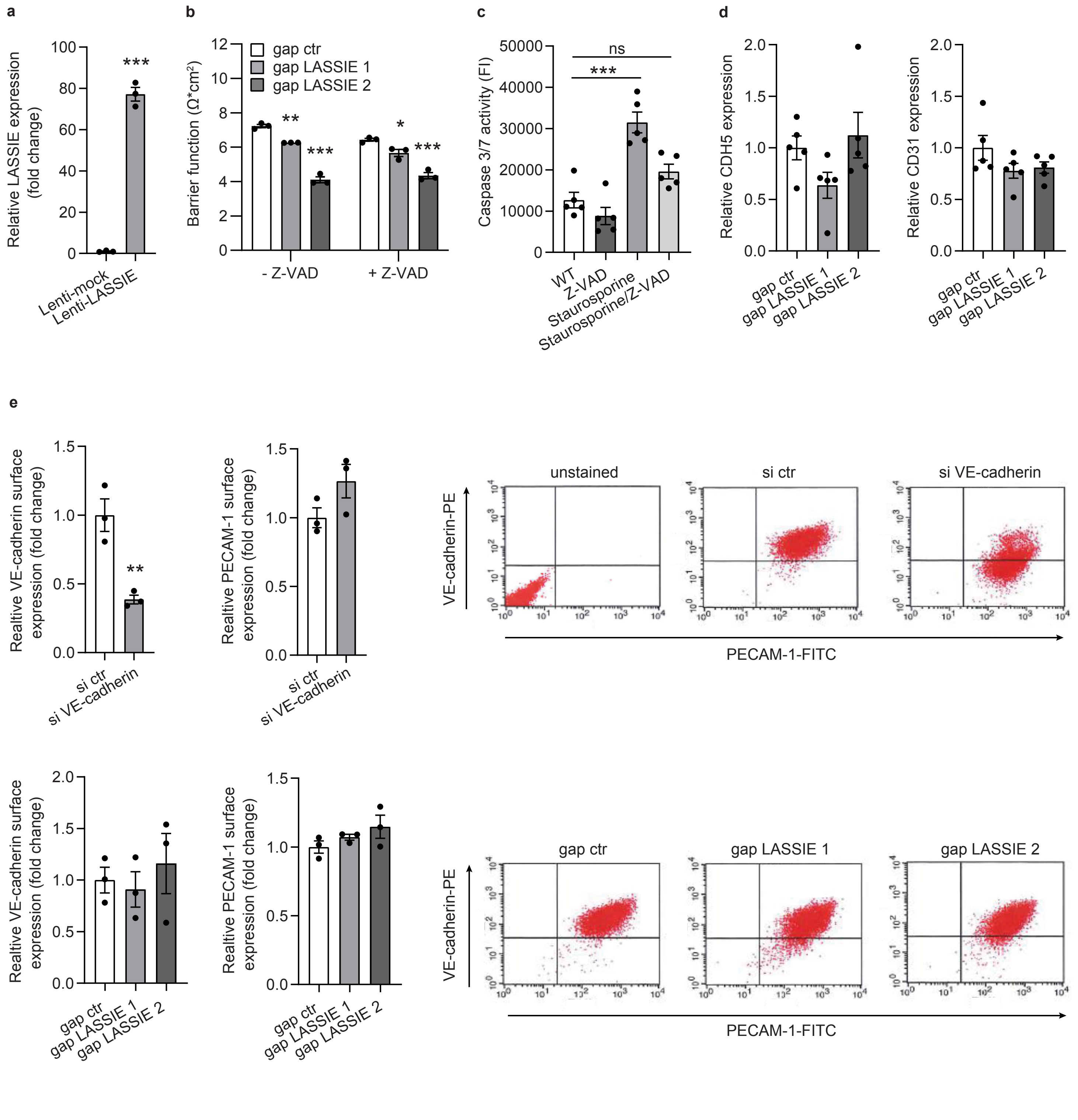

Supplementary Figure 5. (a) Lentiviral-mediated (Lenti) overexpression of LASSIE in HUVECs was analyzed by qRT-PCR. Expression is relative to mock-transduced cells and normalized to RPLP0 mRNA (n=3; Unpaired t test; p=0.000021). **(b)** The effect of GapmeR-mediated (gap) silencing of LASSIE on HUVECs barrier integrity was assessed by ECIS at 400 Hz. HUVECs were treated with the pan-caspase inhibitor Z-VAD-FMK (50 μM) to analyze the effect of apoptosis inhibition on LASSIE-mediated barrier integrity, 72 h post transfection (n=3; 1-way ANOVA; -Z-VAD-FMK: gap ctr vs. gap LASSIE 1: p=0.0018; gap ctr vs. gap LASSIE 2: p<0.0001; +Z-VAD-FMK: gap ctr vs. gap LASSIE 1: p=0.0307; gap ctr vs. gap LASSIE 2: p=0.0002). **(c)** Apoptosis induction of untreated/Staurosporine (200nM)/Z-VAD-FMK (50 μM) wild type (WT) HUVECs was assessed by caspase-3/7 activity, measuring fluorescence intensity (FI) (n=5; 1-way ANOVA; WT vs. Staurosporine: p<0.0001). **(d-e)** HUVECs were transfected with anti-LASSIE or control (ctr) GapmeR (gap), subsequent experiments were performed 48 h after transfection. **(d)** CD31 and CDH5 RNA expression was assessed by qRT-PCR, expression values were normalized to RPLP0 mRNA (n=5; 1-way ANOVA). **(e)** Cell surface expression of PECAM-1 and VE-cadherin was determined by flow cytometry, anti-VE-cadherin siRNA (si) treated cells are shown as positive control. Expression values are relative to control cells (n=3; paired t test: sictr vs. siVE-cadherin: p=0.0075). (*p<0.05; **p<0.01; ***p<0.001)

Supplementary Figure 6

a

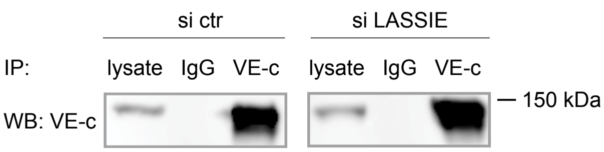

b

gene name	Protein name	fold enriched	
		LASSIE-AS purification	VE-cadherin Co-IP
HSPA9	Stress-70 protein, mitochondrial	6.19	0.39
HSPA6	Heat shock 70 kDa protein 6	6.57	0.67
EPB41L3	Band 4.1-like protein 3	5.64	0.42
TUBA1B; TUBA4A	Tubulin alpha-1B/4A chain	2.1	0.62
NES	Nestin	4.23	0.42

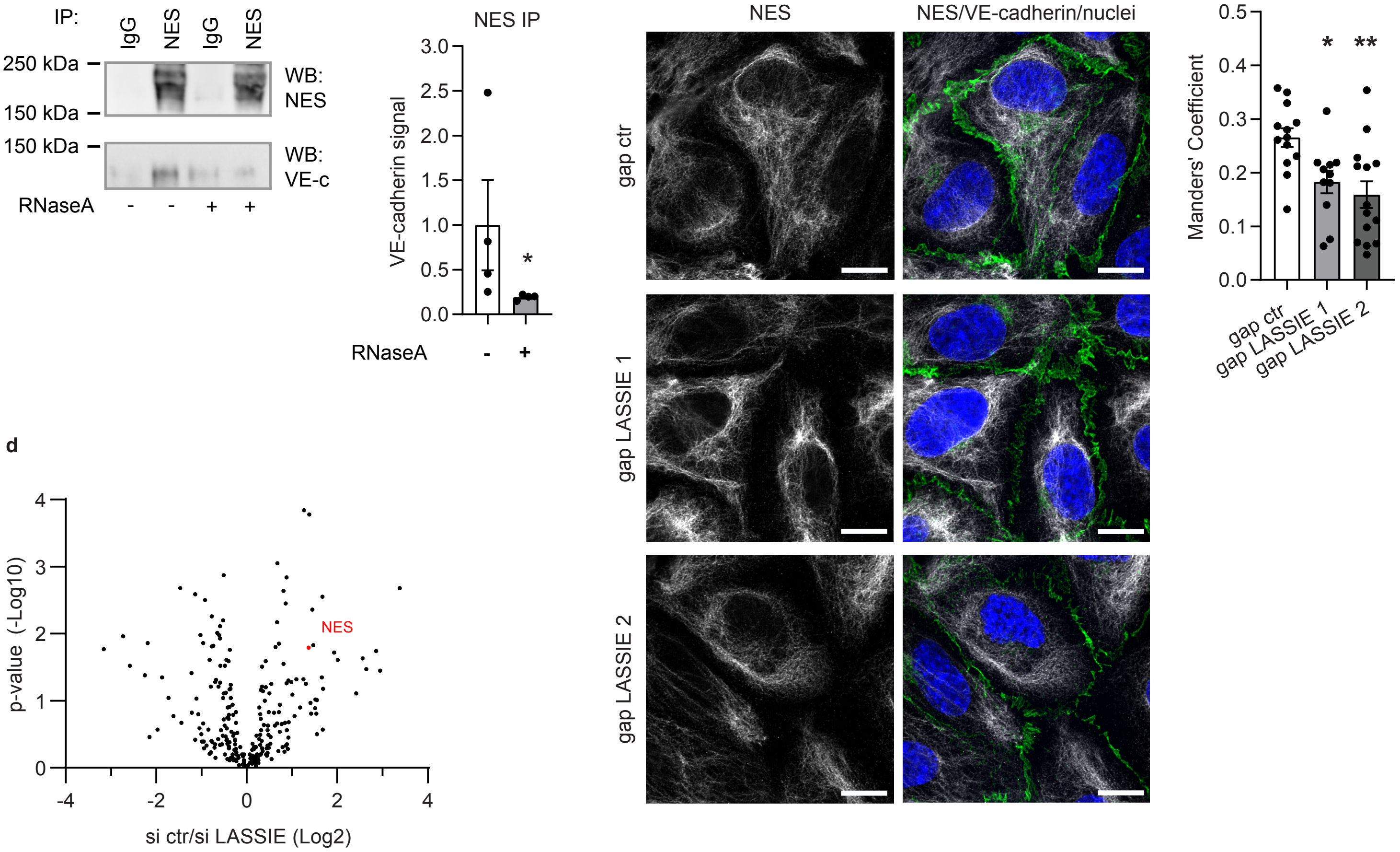

Supplementary Figure 6. (a) HUVEC cell lysates of anti-LASSIE or control (ctr) siRNA (si) treated cells were used for Immunoprecipitation (IP) by incubation with an anti-VE-cadherin (VE-c) antibody. IP efficiency was analyzed by Western Blotting (WB), 1 % of the lysate was used as a control. **(b)** Significantly enriched proteins by LASSIE anti-sense (AS) purification and VE-cadherin Co-Immunoprecipitation (Co-IP) of anti-LASSIE or control (ctr) siRNA (si) treated HUVEC cell lysates, identified by mass spectrometry were compared. Overlapping proteins from both data sets are listed. LFQ value ratios of the significantly enriched proteins are depicted (LASSIE-AS vs. ctr oligo for LASSIE-AS purification and si LASSIE vs. si ctr for VE-cadherin Co-Immunoprecipitation). **(c)** The involvement of RNAs in nestin (NES) - VE-cadherin (VE-c) interaction was analyzed by Co-Immunoprecipitation (IP). HUVEC cell lysates were treated with RNaseA and were used for IP by incubation with an anti-NES antibody. IP efficiency and co-IP of VE-c was analyzed by Western Blotting (WB), band intensities were determined using ImageQuantTL (n=4; Mann Whitney test; p=0.0286). **(d)** RNA -antisense purification was performed in control (si ctr) and LASSIE-silenced (si LASSIE) HUVECs, proteins were captured in both conditions with an anti-LASSIE oligonucleotide. Biotin elutions were analyzed by Mass spectrometry (n=5). Volcano plot depicts captured proteins. **(e-f)** HUVECs were treated with anti-LASSIE or control (ctr) GapmeR (gap) and immuno-stained for VE-cadherin (green) and nestin (NES; white). Nuclei were stained with DAPI (blue). **(e)** Representative images are shown. Scale bars are 10 µm. **(f)** Co-localization of VE-cadherin and nestin was quantified by Manders' Coefficient using ImageJ and JACoP plugin (n=13 (gap ctr) n=11 (gap LASSIE 1) n=14 (gap LASSIE 2) over two different experiments were analyzed; 1-way ANOVA; gap ctr vs. gap LASSIE 1: p=0.0246; gap LASSIE 2: p=0.0020). (*p<0.05; **p<0.01; ***p<0.001)

Supplementary Figure 7

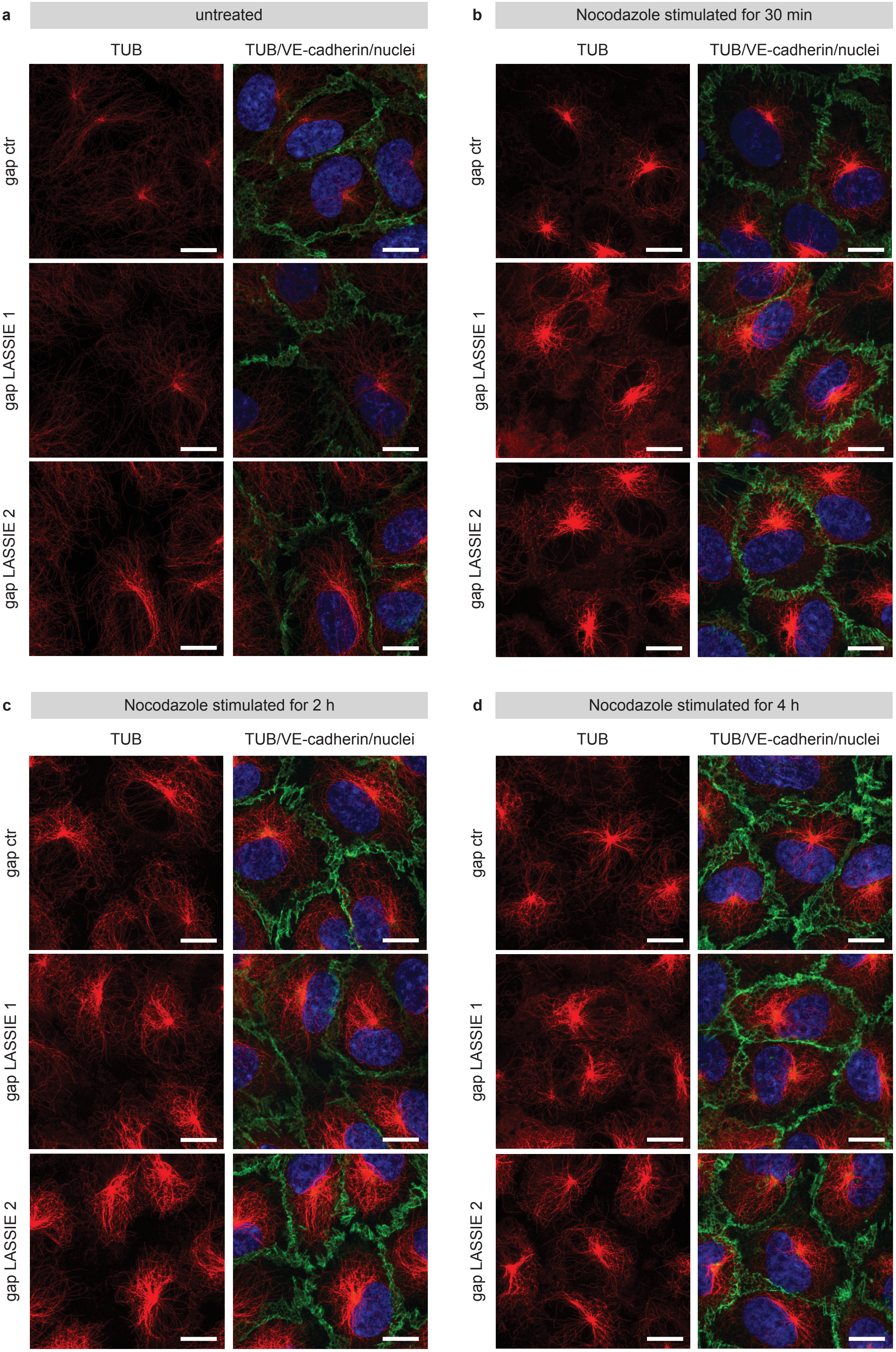

Supplementary Figure 7. (a-d) HUVECs were treated with anti-LASSIE or control (ctr) GapmeR (gap) and immunostained for VE-cadherin (green) and α-Tubulin (red). Nuclei were stained with DAPI (blue). Representative images are shown. Scale bars are 10 µm. **(b-d)** Cells were stimulated with Nocodazole (350 nM) for the indicated time periods.

Supplementary Figure 8

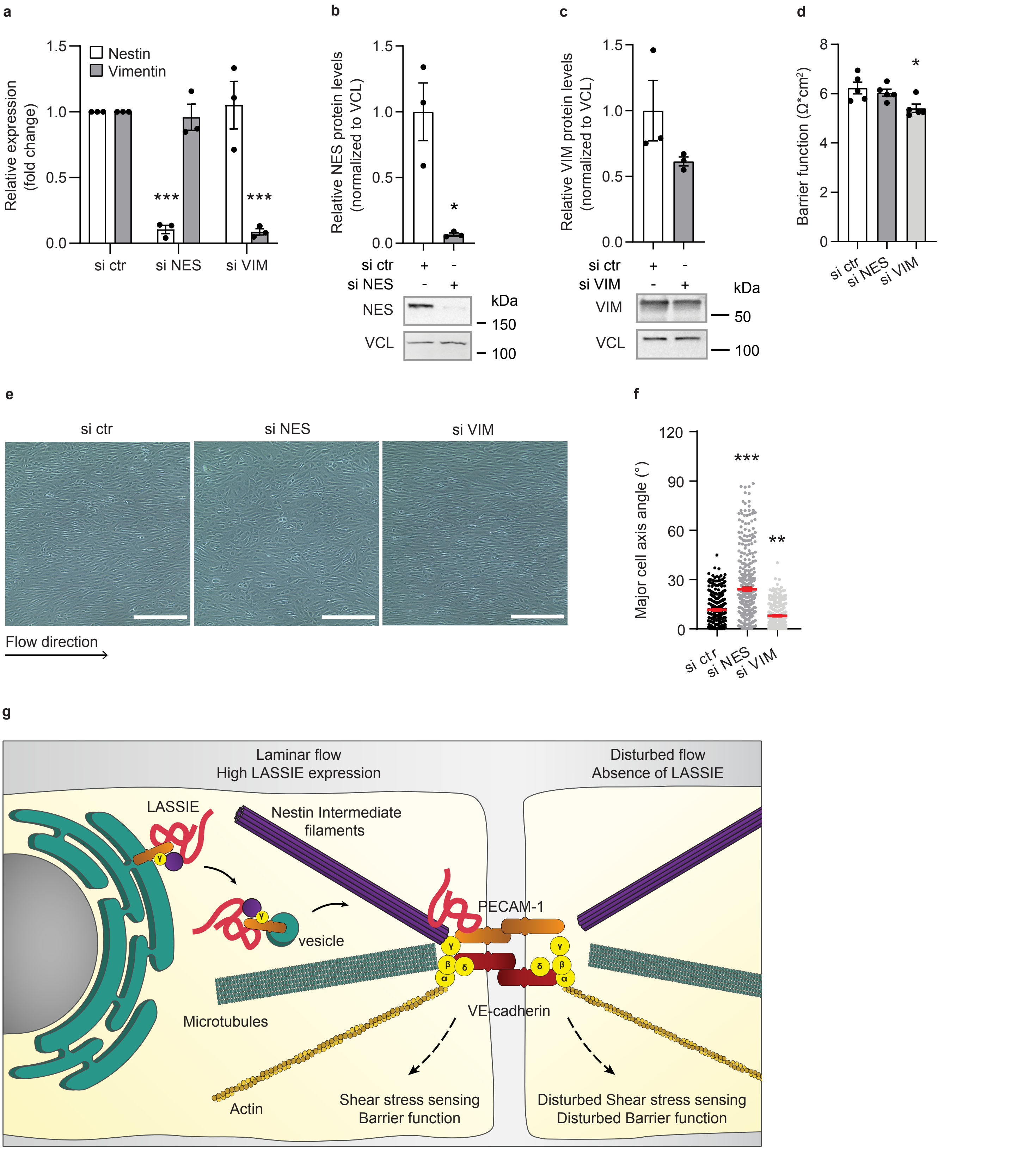

Supplementary Figure 8. (a-f) HUVECs were transfected with siRNA (si) targeting nestin (NES) and vimentin (VIM) or a respective control sequence (ctr). **(a)** Relative expression of nestin and vimentin was determined by qRT-PCR, shown relative to control cells and normalized to RPLP0 expression (n=3; 2-way ANOVA; Nestin: si ctr vs. si NES: p<0.0001; Vimentin: si ctr vs. si VIM: p<0.0001). **(b-c)** Relative expression of nestin (NES) and vimentin (VIM) was determined by Western Blotting. Band intensities were determined using ImageQuantTL and normalized to vinculin (VCL) expression (n=3; Unpaired t test; si ctr vs. si NES: p=0.0132). **(d)** Barrier integrity was assessed by Electrical Cell-Substrate Impedance Sensing (ECIS) at 400 Hz, 72 h post transfection (n=5; 1-way ANOVA; si ctr vs. si VIM: p=0.0227). **(e-f)** HUVECs were exposed to laminar shear stress (20 dyn/cm² for 48 h). **(e)** Representative bright field images are shown. Scale bars are 400 µm. **(f)** Shear stress-induced cell alignment was quantified by determining the angle of the major cell axis; n=414 (si ctr) n=412 (si NES) n=413 (siVIM) over two independent experiments (1-way ANOVA; si ctr vs. si NES: p<0.0001; si ctr vs. si VIM: p<0.0001). **(g)** Schematic model of the proposed LASSIE mechanism. LASSIE is localized at the endoplasmic reticulum facilitating the interaction of junctional and cytoskeletal intermediate filament proteins. That complex is translocated to the membrane where it is necessary for normal barrier function and shear stress sensing. (*p<0.05; **p<0.01; ***p<0.001)

Supplementary Table 1: Sequences of oligonucleotides used in this study.

**Primers used in qRT-PCR analysis**	**Sequence (5'-3')**
GAPDH	ATGGAAATCCCATCACCATCTT<br>CGCCCCACTTGATTTTGG
RPLP0	TCGACAATGGCAGCATCTAC<br>ATCCGTCTCCACAGACAAGG
RPL27	ATCGCCAAGAGATCAAAGATAA<br>TCTGAAGACATCCTTATTGACG
HPRT1	GCTATAAATTCTTTGCTGACCTGCTG<br>AATTACTTTTATGTCCCCTGTTGACTGG
LASSIE	TCTTGCCAGAGTGTCCTTCA<br>GTGCTCCTCCTTCACCTTCT
KLF2	CAAGACCTACACCAAGAGTTCG<br>CATGTGCCGTTTCATGTGC
Malat1	GTGATGCGAGTTGTTCTCCG<br>CTGGCTGCCTCAATGCCTAC
ICAM	ATGCCCAGACATCTGTGTCC<br>TCCTTTTTAGGCAACGGGGT
VCAM	GGGAAGCCGATCACAGTCAA<br>CTCCAGCCTGTCAAATGGGT
Nestin	CAGGAGAAACAGGGCCTACA<br>TGGGAGCAAAGATCCAAGAC
Vimentin	ACGTCTTGACCTTGAACGCA<br>CGTGAGGTCAGGCTTGGAAA
CD31	TGACCCTTCTGCTCTGTTCAA<br>CTTCCCATTTTGCACCGTCC
CDH5	CGGCGCCAAAAGAGAGATTG<br>CACGCTTGACTTGATCTTGCC
zebrafish elf1a	CTTCTCAGGCTGACTGTGC<br>CCGCTAGCATTACCCTCC
zebrafish BC091967	GTAGTGAGGAAAGGTGAGACAC<br>GGTCCTTTGTTGTGCGATGA
zebarfish KLF2a	ACCTATTGCTTGTAGCTGGTT

zebrafish KLF2a	TAAAAGGCACTGTCGTGATG
**Primers used in RNaseH asseccibility assay**	
LASSIE	TCTTGCCAGAGTGTCCTTCA<br>GTGCTCCTCCTTCACCTTCT
RNaseH test	GGCTTGATGGGGAGAAGGAAG<br>AAACCCCGAGCCAAACGTAT

**Primers used in 5´ and 3´ RACE**	
LASSIE 1 - 3' RACE	CCTCTGACCACTCGACCAGCCCAGC
LASSIE 2 - 3' RACE	GCCATGATTGTGAGGCCTCCACAGCC
LASSIE 1 - 5' RACE	TCTTCCCAGGAGTCCTTGCCACCAA
LASSIE 2 - 5' RACE	ACAGGCTTGATGGGGAGAAGGAAGA
**DNA oligos used in RNase H accessibility assay**	
DNA LASSIE 1	GAGTCCTTGCCACCAA
pd oligo LASSIE	CAGAAATGTACACCATGCTGG
**2′O-Me-RNA probes used in RNA pulldown**	
ctr	mGmCmAmAmGmGmAmAmCmGmUmGmUmGmAmGmAmCmUmA/iSp9/rArCrGrArUrC/3deSBioTEG
LASSIE	mCmAmGmAmAmAmUmGmUmAmCmAmCmCmAmU mGmCmUmGmG/iSp9/rArCrGrArUrC/3deSBioTEG
**LNA™ GapmeRs**	
gap ctr (negative control A)	LNA™ longRNA GapmeR, Negative control A (300610)
gap LASSIE 1	GAGTCCTTGCCACCAA
gap LASSIE 2	AAGATAAAGGCAATGC
**siRNAs**	
si ctr	MISSION siRNA Universal Negative Cotnrol #1 (SIC001)
si LASSIE 1	CUUGCCACCAAUCUCUCUG
si LASSIE 2	AAGAUAAAGGCAAUGCUUG
si VE-cadherin	NM_001795, siRNA ID: SASI_Hs01_00100751
si Vimentin	SASI_Hs01_00044035
si Nestin	SASI_Hs01_00097739
**morpholinos**	
zebrafish ctr MO	CCTCTTACCTCAGTTACAATTTATA
zebrafish Tnnt2 MO	CATGTTTGCTCTGATCTGACACGCA

Supplementary Table 2: Antibodies used in this study.

anti-human antibody	Company	Catalog #	Species	Clone	Dilution	Application
Nestin	Abcam	ab6320	mouse	196908	2.5 µg	IP
PECAM-1	R&D SYSTEMS	BBA7	mouse	9G11	5 µg	IP
VE-cadherin	Cell Signaling	2500	rabbit	D87F2	1:50	IP
Normal rabbit IgG	Cell Signaling	2729S	rabbit		5 µg	IP
Normal mouse IgG	Merck Millipore	12-371	mouse		5 / 2.5 µg	IP
Calreticulin	Abcam	ab22683	mouse	FMC 75	1:400	IF
β-Catenin	Sigma Aldrich	C7082	mouse	6F9	1:1000	IF/PLA
γ-Catenin	MyBiosource.com	MBS470014	mouse		1:100	IF
Nestin	Abcam	ab6320	mouse	196908	1:400	IF/PLA
PECAM-1	BD Biosciences	550389	mouse	WM59	1:50	IF
PECAM-1	Santa Cruz Biotechnology	sc-1506	goat		1:100	IF
VE-cadherin	Cell Signaling	2500	rabbit	D87F2	1:400	IF/PLA
TRF1	Abcam	ab10579	mouse	TRF-78	1:100	IF/PLA
α-Tubulin	Abcam	ab7291	mouse	DM1A	1:400	IF
FITC Mouse Anti-Human CD31	BD Biosciences	555445	mouse	WM59	1:20	Flow cytometry
PE Mouse anti-Human CD144	BD Biosciences	561714	mouse	55-7H1	1:20	Flow cytometry
FITC Mouse IgG1, κ Isotype Control	BD Biosciences	555748	mouse	MOPC-21	1:20	Flow cytometry
PE Mouse IgG1, κ Isotype Control	BD Biosciences	555749	mouse	MOPC-21	1:20	Flow cytometry
Nestin	Abcam	ab6320	mouse	196908	1:1000	WB
PECAM-1	R&D SYSTEMS	BBA7	mouse	9G11	1:500	WB
VE-cadherin	Sigma Aldrich	V1514	rabbit		1:1000	WB
Vinculin	Santa Cruz Biotechnology	sc-5573	rabbit		1:500	WB
Vimentin	Thermo Fisher Scientific	PA5-27231	rabbit		1:2000	WB

Supplementary Table 3: Sequence of LASSIE transcript variant 1.

LncRNA transcript variants were identified by 5´ and 3´ RACE. RNA of KLF2 overexpressing HUVECs was used as a template. The sequence of the most abundant LASSIE transcript variant is listed.

**LASSIE transcript variant 1 (5'- 3')**
ACCGGGTCTTGCCTTCTGCCATGTAAGATATGACTGTGCTCCTCCTTCACCTTCTGCCATGATTGTGAGGCCTCCACAGCCATG<br>TGGAACTGGAAGCTGTTTTTCCCTCTTTGGGGTCTCAGAGAGATTGGTGGCAAGGACTCCTGGGAAGAGAAAAGCTGAAGGAC<br>ACTCTGGCAAGAGGAAACCCCGAGCCAAACGTATGGCCACCTCTGACCACTCGACCAGCCCAGCATGGTGTACATTTCTGGGT<br>AGCTTATCACGCCTTCCAAGCATTGCCTTTATCTTCCTTCTCCCCATCAAGCCTGTATTGTTTTCCTTTTGAATCATAAGCACTTTT<br>GTTAAATACCTCAAATCTTTCGAGAACAGCAAGGAGCAAAATAAACTTAACTTCTC
