# Supplementary material for: Long non-coding RNA LASSIE regulates shear stress sensing and endothelial barrier function
Source: Commun Biol. 2020 May 26;3:265. doi: 10.1038/s42003-020-0987-0 (PMC7251106; doi:10.1038/s42003-020-0987-0)
Supplement: Supplementary file 2 — Description of Additional Supplementary Files [file 42003_2020_987_MOESM2_ESM.pdf]

Supplementary data 1: LASSIE anti-sense purified proteins identified by Mass spectromerty.

Endogenous LASSIE-protein complexes were captured by RNA-antisense (AS) purification using an anti-LASSIE desthiobiotin-coupled 2′O-Me-RNA and a control oligonucleotide. Purified proteins were analysed by Mass spectrometry, fold change of respective LFQ values of the significantly enriched proteins ($p<0.05$) are listed ($n=5$).

Supplementary data 2: GO term analysis of LASSIE interacting proteins.

Endogenous LASSIE-protein complexes were captured by RNA-antisense purification and analysed by Mass spectrometry. Significantly enriched proteins ($p < 0.05$) were used for Gene Ontology (GO) term pathway analysis. The top 20 GO terms are listed. “Count” indicates the number of regulated genes that are present in the pathway.

Supplementary data 3: VE-cadherin associated proteins in the absence of LASSIE, identified by Mass spectrometry.

Cell lysates of anti-LASSIE or control (ctr) siRNA treated HUVECs were used for VE-cadherin Immunoprecipitation. Captured proteins were analyzed by Mass spectrometry (MS). LFQ ratios of si LASSIE ($n=4$) to si ctr ($n=5$) and the IBAQ values (sequence coverage >4%) of the significantly changed proteins are listed.

Supplementary data 4: LASSIE-AS purified proteins identified by Mass spectrometry of control and LASSIE silenced cells.

Endogenous LASSIE-protein complexes were captured by RNA-antisense (AS) purification using an anti-LASSIE desthiobiotin-coupled 2′O-Me-RNA oligonucleotide in HUVECs treated with siRNA (si) targeting LASSIE or a control (ctr) sequence. Purified proteins were analysed by Mass spectrometry, fold change of respective LFQ values of the significantly enriched proteins ($p<0.05$) are listed ($n=5$).

Supplementary data 5: Source data underlying all experiments.

The raw data from the experiments in this study are gathered in the spreadsheet.
