# Supplementary material for: Long non-coding RNA LASSIE regulates shear stress sensing and endothelial barrier function
Source: Commun Biol. 2020 May 26;3:265. doi: 10.1038/s42003-020-0987-0 (PMC7251106; doi:10.1038/s42003-020-0987-0)
Supplement: Supplementary file 8 — Reporting Summary [file 42003_2020_987_MOESM8_ESM.pdf]

Corresponding author(s): DBPR NCOMMS-19-02999A-Z

Last updated by author(s): Feb 6, 2020

Reporting Summary

Nature Research wishes to improve the reproducibility of the work that we publish. This form provides structure for consistency and transparency in reporting. For further information on Nature Research policies, see Authors & Referees and the Editorial Policy Checklist.

Statistics

For all statistical analyses, confirm that the following items are present in the figure legend, table legend, main text, or Methods section.

n/a	Confirmed	
☐	☒	The exact sample size (*n*) for each experimental group/condition, given as a discrete number and unit of measurement
☐	☒	A statement on whether measurements were taken from distinct samples or whether the same sample was measured repeatedly
☐	☒	The statistical test(s) used AND whether they are one- or two-sided<br>*Only common tests should be described solely by name; describe more complex techniques in the Methods section.*
☐	☒	A description of all covariates tested
☐	☒	A description of any assumptions or corrections, such as tests of normality and adjustment for multiple comparisons
☐	☒	A full description of the statistical parameters including central tendency (e.g. means) or other basic estimates (e.g. regression coefficient) AND variation (e.g. standard deviation) or associated estimates of uncertainty (e.g. confidence intervals)
☐	☒	For null hypothesis testing, the test statistic (e.g. *F*, *t*, *r*) with confidence intervals, effect sizes, degrees of freedom and *P* value noted<br>*Give P values as exact values whenever suitable.*
☒	☐	For Bayesian analysis, information on the choice of priors and Markov chain Monte Carlo settings
☒	☐	For hierarchical and complex designs, identification of the appropriate level for tests and full reporting of outcomes
☒	☐	Estimates of effect sizes (e.g. Cohen's *d*, Pearson's *r*), indicating how they were calculated

Our web collection on statistics for biologists contains articles on many of the points above.

Software and code

Policy information about availability of computer code

Data collection	*Provide a description of all commercial, open source and custom code used to collect the data in this study, specifying the version used OR state that no software was used.*
Data analysis	GraphPad Prism 7

For manuscripts utilizing custom algorithms or software that are central to the research but not yet described in published literature, software must be made available to editors/reviewers. We strongly encourage code deposition in a community repository (e.g. GitHub). See the Nature Research guidelines for submitting code & software for further information.

Data

Policy information about availability of data

All manuscripts must include a data availability statement. This statement should provide the following information, where applicable:

- Accession codes, unique identifiers, or web links for publicly available datasets
- A list of figures that have associated raw data
- A description of any restrictions on data availability

All data generated and analysed during this study are available from the corresponding author on reasonable request. Microarray data are deposited in the Gene Expression Omnibus repository under the accession number GSE146110. Proteomics data are deposited in the PRIDE archive under the accession numbers PXD018724, PXD018734 and PXD018725. Source data are provided as Supplementary Data 5.

Field-specific reporting

Please select the one below that is the best fit for your research. If you are not sure, read the appropriate sections before making your selection.

☒ Life sciences ☐ Behavioural & social sciences ☐ Ecological, evolutionary & environmental sciences

For a reference copy of the document with all sections, see nature.com/documents/nr-reporting-summary-flat.pdf

Life sciences study design

All studies must disclose on these points even when the disclosure is negative.

Sample size	Samples sizes were determined using power calculation
Data exclusions	Significant outliers were identified using Grubbs' outlier test and excluded from analysis.
Replication	All results were reproduced in at least 3 technically independent replicates.
Randomization	*Describe how samples/organisms/participants were allocated into experimental groups. If allocation was not random, describe how covariates were controlled OR if this is not relevant to your study, explain why.*
Blinding	All researchers involved in animal experiments were blinded to the treatment during data collection and analysis.

Reporting for specific materials, systems and methods

We require information from authors about some types of materials, experimental systems and methods used in many studies. Here, indicate whether each material, system or method listed is relevant to your study. If you are not sure if a list item applies to your research, read the appropriate section before selecting a response.

Materials & experimental systems

n/a	Involved in the study	
☐	☒	Antibodies
☐	☒	Eukaryotic cell lines
☒	☐	Palaeontology
☐	☒	Animals and other organisms
☒	☐	Human research participants
☒	☐	Clinical data

Methods

n/a	Involved in the study	
☒	☐	ChIP-seq
☐	☒	Flow cytometry
☒	☐	MRI-based neuroimaging

Antibodies

Antibodies used

Nestin Abcam ab6320 mouse 196908 2.5 µg IP
PECAM-1 R&D SYSTEMS BBA7 mouse 9G11 5 µg IP
VE-cadherin Cell Signaling 2500 rabbit D87F2 1:50 IP
Normal rabbit IgG Cell Signaling 2729S rabbit 5 µg IP
Normal mouse IgG Merck Millipore 12-371 mouse 5 / 2.5 µg IP
Calreticulin Abcam ab22683 mouse FMC 75 1:400 IF
β-Catenin Sigma Aldrich C7082 mouse 6F9 1:1000 IF/PLA
γ-Catenin MyBiosource.com MBS470014 mouse 1:100 IF
Nestin Abcam ab6320 mouse 196908 1:400 IF/PLA
PECAM-1 BD Biosciences 550389 mouse WM59 1:50 IF
PECAM-1 Santa Cruz Biotechnology sc-1506 goat 1:100 IF
VE-cadherin Cell Signaling 2500 rabbit D87F2 1:400 IF/PLA
TRF1 Abcam ab10579 mouse TRF-78 1:100 IF/PLA
α-Tubulin Abcam ab7291 mouse DM1A 1:400 IF
FITC Mouse Anti-Human CD31 BD Biosciences 555445 mouse WM59 1:20 Flow cytometry
PE Mouse anti-Human CD144 BD Biosciences 561714 mouse 55-7H1 1:20 Flow cytometry
FITC Mouse IgG1, κ Isotype Control BD Biosciences 555748 mouse MOPC-21 1:20 Flow cytometry
PE Mouse IgG1, κ Isotype Control BD Biosciences 555749 mouse MOPC-21 1:20 Flow cytometry
Nestin Abcam ab6320 mouse 196908 1:1000 WB
PECAM-1 R&D SYSTEMS BBA7 mouse 9G11 1:500 WB
VE-cadherin Sigma Aldrich V1514 rabbit 1:1000 WB
Vinculin Santa Cruz Biotechnology sc-5573 rabbit 1:500 WB
Vimentin Thermo Fisher Scientific PA5-27231 rabbit 1:2000 WB

Validation	*Describe the validation of each primary antibody for the species and application, noting any validation statements on the manufacturer's website, relevant citations, antibody profiles in online databases, or data provided in the manuscript.*

Eukaryotic cell lines

Policy information about cell lines

Cell line source(s)	Primary cells:<br>HUVECs were purchased from Lonza. MVEC were isolated from pleura-free peripheral lung tissues and PAECs from rings of the arteria pulmonalis.<br>Cell lines:<br>HEK293T cells were purchased from ATCC.
Authentication	The cell lines were not authenticated.
Mycoplasma contamination	All cells were tested negative for mycoplasma contamination.
Commonly misidentified lines<br>(See ICLAC register)	*Name any commonly misidentified cell lines used in the study and provide a rationale for their use.*

Animals and other organisms

Policy information about studies involving animals; ARRIVE guidelines recommended for reporting animal research

Laboratory animals	Zebrafish
Wild animals	The study did not involve wild animals.
Field-collected samples	The study did not involve samples collected from the field.
Ethics oversight	All animal procedures conformed to EU Directive 86/609/EEC and Recommendation 2007/526/EC regarding the protection of animals used for experimental and other scientific purposes

Note that full information on the approval of the study protocol must also be provided in the manuscript.

Flow Cytometry

Plots

Confirm that:

☒ The axis labels state the marker and fluorochrome used (e.g. CD4-FITC).

☒ The axis scales are clearly visible. Include numbers along axes only for bottom left plot of group (a 'group' is an analysis of identical markers).

☐ All plots are contour plots with outliers or pseudocolor plots.

☒ A numerical value for number of cells or percentage (with statistics) is provided.

Methodology

Sample preparation	HUVECs were stained using Annexin V and 7-AAD or VE-Cadherin and PECAM-1
Instrument	BD FACSCanto II machine (BD Biosciences) and FACSCalibur™ device (BD Biosciences)
Software	BD FACSDiva™ software and FlowJo
Cell population abundance	*Describe the abundance of the relevant cell populations within post-sort fractions, providing details on the purity of the samples and how it was determined.*
Gating strategy	*Describe the gating strategy used for all relevant experiments, specifying the preliminary FSC/SSC gates of the starting cell population, indicating where boundaries between "positive" and "negative" staining cell populations are defined.*

☒ Tick this box to confirm that a figure exemplifying the gating strategy is provided in the Supplementary Information.
