# Supplementary material for: Long non-coding RNA LASSIE regulates shear stress sensing and endothelial barrier function
Source: Commun Biol. 2020 May 26;3:265. doi: 10.1038/s42003-020-0987-0 (PMC7251106; doi:10.1038/s42003-020-0987-0)
Supplement: Supplementary file 9 — Peer Review File [file 42003_2020_987_MOESM9_ESM.pdf]

Editorial Note: *This manuscript has been previously reviewed at another Nature Research journal. The manuscript was considered suitable for publication without further review at Communications Biology*
